# Supplementary material for: Development of a core outcome set and identification of patient-reportable outcomes for primary brain tumour trials: protocol for the COBra study
Source: BMJ Open. 2022 Sep 30;12(9):e057712. doi: 10.1136/bmjopen-2021-057712 (PMC9528585; doi:10.1136/bmjopen-2021-057712)
Supplement: Supplementary data [file bmjopen-2021-057712supp001.pdf]

Appendix 1

		Page
Title 1a	Identify in the title that the paper describes the protocol for the planned development of a COS	1
Abstract 1b	Provide a structured abstract	2
INTRODUCTION Background and objectives 2a	Describe the background and explain the rationale for developing the COS, and identify the reasons why a COS is needed and the potential barriers to its implementation	3-5, 11
2b	Describe the specific objectives with reference to developing a COS	5
Scope 3a	Describe the health condition(s) and population(s) that will be covered by the COS	5
3b	Describe the intervention(s) that will be covered by the COS	5
3c	Describe the context of use for which the COS is to be applied	5
METHODS Stakeholders 4	Describe the stakeholder groups to be involved in the COS development process, the nature of and rationale for their involvement and also how the individuals will be identified; this should cover involvement both as members of the research team and as participants in the study	7-10
Information sources 5a	Describe the information sources that will be used to identify the list of outcomes. Outline the methods or reference other protocols/papers	6-10
5b	Describe how outcomes may be dropped/ combined, with reasons	9-10
Consensus process 6	Describe the plans for how the consensus process will be undertaken	10
Consensus definition 7a	Describe the consensus definition	10
7b	Describe the procedure for determining how outcomes will be added/combined/dropped from consideration during the consensus process	10
ANALYSIS Outcome scoring/ feedback 8	Describe how outcomes will be scored and summarised, describe how participants will receive feedback during the consensus process	10
Missing data 9	Describe how missing data will be handled during the consensus process	10
ETHICS and DISSEMINATION Ethics approval/ informed consent 10	Describe any plans for obtaining research ethics committee/institutional review board approval in relation to the consensus process and describe how informed consent will be obtained (if relevant)	9-11
Dissemination 11	Describe any plans to communicate the results to study participants and COS users, inclusive of methods and timing of dissemination	11
ADMINISTRATIVE INFORMATION Funders 12	Describe sources of funding, role of funders	14
Conflicts of interest 13	Describe any potential conflicts of interest within the study team and how they will be managed	14
