# Supplementary material for: Development of a core outcome set and identification of patient-reportable outcomes for primary brain tumour trials: protocol for the COBra study
Source: BMJ Open. 2022 Sep 30;12(9):e057712. doi: 10.1136/bmjopen-2021-057712 (PMC9528585; doi:10.1136/bmjopen-2021-057712)
Supplement: Supplementary data [file bmjopen-2021-057712supp002.pdf]

Appendix 2

1. ClinicalTrials.gov
Condition/Disease: glioma OR astrocytoma OR oligodendroglioma OR oligoastrocytoma OR ependymoma OR astroblastoma OR anaplastic gangioglioma OR glioblastoma OR GBM OR Glioblastoma multiforme
Study type: Interventional Studies (Clinical Trials)
Age: Adult 18-64 AND Older Adult (65+)
Phase: III

2. ISRCTN.com
Each term searched individually:
Condition/Disease: glioma; astrocytoma; oligodendroglioma; oligoastrocytoma; ependymoma; astroblastoma; anaplastic gangioglioma; glioblastoma; GBM; Glioblastoma multiforme
