# Supplementary material for: Development of a core outcome set and identification of patient-reportable outcomes for primary brain tumour trials: protocol for the COBra study
Source: BMJ Open. 2022 Sep 30;12(9):e057712. doi: 10.1136/bmjopen-2021-057712 (PMC9528585; doi:10.1136/bmjopen-2021-057712)
Supplement: Author's manuscript [file bmjopen-2021-057712.draft_revisions.pdf]

BMJ Open

BMJ Open is committed to open peer review. As part of this commitment we make the peer review history of every article we publish publicly available.

When an article is published we post the peer reviewers' comments and the authors' responses online. We also post the versions of the paper that were used during peer review. These are the versions that the peer review comments apply to.

The versions of the paper that follow are the versions that were submitted during the peer review process. They are not the versions of record or the final published versions. They should not be cited or distributed as the published version of this manuscript.

BMJ Open is an open access journal and the full, final, typeset and author-corrected version of record of the manuscript is available on our site with no access controls, subscription charges or pay-per-view fees (http://bmjopen.bmj.com).

If you have any questions on BMJ Open's open peer review process please email info.bmjopen@bmj.com

BMJ Open

Development of a Core Outcome Set and Identification of Patient-Reportable Outcomes for Primary Brain Tumour Trials: Protocol for the COBra Study

Journal:	*BMJ Open*
Manuscript ID	bmjopen-2021-057712
Article Type:	Protocol
Date Submitted by the Author:	27-Sep-2021
Complete List of Authors:	Retzer, Ameeta; University of Birmingham, Centre for Patient Reported Outcomes Research, Institute for Applied Health Research; National Institute for Health Research, Applied Research Centre, West Midlands<br>Sivell, Stephanie; Cardiff University, Marie Curie Palliative Care Research Centre, Division of Population Medicine, Cardiff University School of Medicine, College of Biomedical and Life Sciences<br>Scott, Hannah; University of Cambridge, Cambridge Public Health, University of Cambridge School of Clinical Medicine<br>Nelson, Annmarie; Cardiff University School of Medicine, Marie Curie Palliative Care Research Centre, Division of Population Medicine, Cardiff University School of Medicine, College of Biomedical and Life Sciences<br>Bulbeck, Helen; brainstrust<br>Seddon, Kathy; Cardiff University<br>Grant, Robin ; University of Edinburgh, Centre for Clinical Brain Sciences<br>Adams, Richard; University of Cardiff, Centre for Trials Research<br>Watts, Colin; University of Birmingham, Institute of Cancer and Genomic Sciences, College of Medical and Dental Sciences<br>Aiyegbusi, Olalekan Lee; University of Birmingham, Centre for Patient Reported Outcomes Research, Institute of Applied Health Research; University of Birmingham, NIHR Birmingham Biomedical Research Centre, University of Birmingham and University Hospitals Birmingham NHS Foundation Trust<br>Kearns, Pamela; University of Birmingham, Institute of Cancer and Genomic Sciences, College of Medical and Dental Sciences; University of Birmingham, NIHR Surgical Reconstruction and Microbiology Research Centre<br>Cruz Rivera, Samantha; University of Birmingham, Centre for Patient Reported Outcomes Research, Institute of Applied Health Research; University of Birmingham, Birmingham Health Partners Centre for Regulatory Science and Innovation<br>Dirven, Linda; Leiden University, Department of Neurology, Leiden University Medical Center; Medical Centre Haaglanden, Department of Neurology<br>Baddeley, Elin; Cardiff University, Marie Curie Palliative Care Research Centre, Division of Population Medicine, Cardiff University School of Medicine, College of Biomedical and Life Sciences<br>Calvert , Melanie ; University of Birmingham, Centre for Patient Reported Outcomes Research, Institute of Applied Health Research; National Institute for Health Research, Applied Research Centre West Midlands

Byrne, Anthony; Cardiff University, Marie Curie Palliative Care Research Centre, Division of Population Medicine, Cardiff University School of Medicine, College of Biomedical and Life Sciences

Keywords: Neurological oncology < NEUROLOGY, Neurological oncology < ONCOLOGY, Adult palliative care < PALLIATIVE CARE, QUALITATIVE RESEARCH, Clinical trials < THERAPEUTICS

SCHOLARONE™ Manuscripts

Development of a Core Outcome Set and Identification of Patient-Reportable Outcomes for Primary Brain Tumour Trials: Protocol for the COBra Study

Authors: Ameeta Retzer[1,2,3], Stephanie Sivell[4], Hannah Scott[5], Annmarie Nelson[4], Helen Bulbeck[6], Kathy Seddon[7], Robin Grant[8], Richard Adams[9], Colin Watts[10], Olalekan Lee Aiyegbusi[1,2,3,11,12], Pamela Kearns[10,13], Samantha Cruz Rivera[1,2,11], Linda Dirven[15,16], Elin Baddeley[4], Melanie Calvert[1,2,3,11,12,13, 17], Anthony Byrne[4]

[1]Institute of Applied Health Research, University of Birmingham, Birmingham, UK

[2]Centre for Patient Reported Outcomes Research, Institute of Applied Health Research, University of Birmingham, Birmingham, UK

[3]National Institute for Health Research (NIHR) Applied Research Centre West Midlands, Birmingham, UK

[4] Marie Curie Palliative Care Research Centre, Division of Population Medicine, Cardiff University School of Medicine, College of Biomedical and Life Sciences, UK

[5] Cambridge Public Health, University of Cambridge School of Clinical Medicine, UK

[6] Brainstrust - the brain cancer people, UK

[7]Cardiff University, UK

[8] University of Edinburgh, UK

[9] Centre for Trials Research, Cardiff University, UK

[10] Institute of Cancer and Genomic Sciences, College of Medical and Dental Sciences, University of Birmingham, UK

[11]Birmingham Health Partners Centre for Regulatory Science and Innovation, University of Birmingham, Birmingham, UK

[12]NIHR Birmingham Biomedical Research Centre, University of Birmingham, Birmingham, UK

[13]NIHR Surgical Reconstruction and Microbiology Research Centre, University of Birmingham, Birmingham, UK

[14] Cancer Research UK Clinical Trials Unit (CRCTU), Institute of Cancer and Genomic Sciences, University of Birmingham, UK

For peer review only

For peer review only - http://bmjopen.bmj.com/site/about/guidelines.xhtml

[15] Department of Neurology, Leiden University Medical Center, Leiden, The Netherlands

[16] Department of Neurology, Haaglanden Medical Center, The Hague, The Netherlands

[17]Midlands Health Data Research UK, Birmingham, UK

Correspondence to: Professor Anthony Byrne (anthony.byrne2@wales.nhs.uk)

Abstract:

Introduction
Primary brain tumours, specifically gliomas, are a rare disease group. The disease and treatment negatively impacts on patients and those close to them. The high rates of physical and cognitive morbidity differ from other cancers causing reduced health-related quality of life. Glioma trials using outcomes that allow holistic analysis of treatment benefits and risks enable informed care decisions. Currently, outcome assessment in glioma trials is inconsistent, hindering evidence synthesis. A core outcome set (COS): an agreed minimum set of outcomes to be measured and reported may address this. International initiatives focus on defining core outcomes assessments across brain tumour types. This paper presents a protocol for developing a COS for use in glioma trials, applicable across glioma types involving UK stakeholders, with provision to identify subsets as required. Due to stakeholder interest in data reported from the patient perspective, outcomes from the COS that can be patient-reported will be identified.
Methods and analysis
Stage I: (i) trial registry review to identify outcomes collected in glioma trials and (ii) systematic review of qualitative literature exploring glioma patient and key stakeholder research priorities. Stage II: semi-structured interviews with glioma patients and caregivers. Outcome lists will be generated from Stages I and II. Stage III: study team will remove duplicate items from the outcome lists and ensure accessible terminology for inclusion in the Delphi survey. Stage IV: a two-round Delphi process whereby the outcomes will be rated by key stakeholders. Stage V: a consensus meeting where participants will finalise the COS. The study team will identify the COS outcomes that can be patient-reported. Further research is needed to match patient-reported outcomes to available measures.
Ethics and dissemination
Ethical approval was obtained (REF SMREC 21/59).
Trial and PROSPERO registration
Core Outcome Measures in Effectiveness Trials (https://www.comet-initiative.org/Studies/Details/1793); PROSPERO (CRD42021236979).

Strengths and Limitations

- This study collects original qualitative data to ensure all outcomes prioritised by glioma patients are identified
- Review of trial registries enables comprehensive identification of outcomes used in trials rather than reliance on often incomplete outcome reporting in trial publications. However, use of trial registries means those that are not registered will not be identified

For peer review only - http://bmjopen.bmj.com/site/about/guidelines.xhtml

- Bias may be introduced by inviting qualitative interview participants from Stage II to take part in Delphi; though this encourages familiarity with concepts, enabling meaningful participation in the Delphi
- Qualitative data collected from the UK population may limit international applicability, though this allows exploration of issues that may be specific to UK context and validation of this COS for use in other settings should be explored.

Introduction:

Primary brain tumours, specifically gliomas, are part of a rare disease group[1]. The disease and its treatment have negative effects on patients and those close to them. The high rates of physical and cognitive morbidity differ from other cancers, with significant impact on a wide range of functional domains. Gliomas are the commonest form of primary brain tumour[2], accounting for 80% of malignant brain tumours. Gliomas represent a heterogeneous group of cancers with variable outcome, traditionally graded from I to IV (least to most aggressive) [2]. However, rapid developments in molecular diagnostics have led to refinements in nomenclature, suggesting a more nuanced approach to brain tumours classification[3]. This would acknowledge the spectrum ranging from a variable but slower-progressing course, such as oligodendroglioma or astrocytoma, to fast-growing tumours such as glioblastoma, a particularly aggressive subtype with a median survival of 12 to 15 months and 5% five year survival rate[4].

The poor prognosis of some glioma patients and the high symptom burden has led to a growing emphasis on their quality of survival[5]. Maintaining cognitive function, physical function and other health-related quality of life aspects throughout the disease trajectory are key considerations alongside very modest survival benefits captured through traditional metrics of tumour response and overall or progression-free survival, particularly for patients with aggressive forms of glioma [6]. Therefore, it is important that glioma intervention studies collect a range of data aligned with patient priorities to enable assessment of the net clinical benefit of treatments [7-10]. These data are known as “outcomes”.

Outcomes include traditional measures such as progression-free survival and radiological tumour response but also Clinical Outcome Assessments (COAs). COAs describe how a patient feels, functions, or survives. COAs include Clinician Reported Outcomes (ClinRO), Observer Reported Outcomes (ObsRO), Performance Outcomes (PerfO), and Patient Reported Outcomes (PROs)[11]. PROs assess a range of outcomes including symptoms, functional health, well-being and psychological issues from the patients’ perspective, without interpretation by a clinician or anyone else [12]. When assessing treatments, PROs enable insight into the impact of treatment on patient’s perceived wellbeing where other outcome data that may indicate minimal differences in disease control and survival, potentially influencing patients’ treatment choices [13].

Interpreting the net clinical benefit of treatments requires effective data synthesis and meta-analyses of trial outcomes. This requires consistent use of outcomes, use of appropriate outcome measures, and diligent data capture, analysis and reporting. Inconsistent outcome use is widespread. A significant lack of standard ontology has been found in cancer clinical trials [14] and in brain tumour studies specifically[15]. Moreover,

For peer review only - http://bmjopen.bmj.com/site/about/guidelines.xhtml

selective outcome and missing data reporting is common[16], introducing bias and hindering evidence synthesis. PROs are critical to the comprehensive evaluation of treatment benefits and side effects, and are increasingly used by regulatory authorities. The Food and Drug Administration (FDA) is prioritising a patient-centred approach to drug development[17], a consistent approach to PRO use generally [18], and in cancer clinical trials specifically[19].The European Medicines Agency (EMA) support PRO use to assess drug efficacy and tolerability in informing product approval in cancer[20], consistent with the FDA[21, 22]. Key PROs for use in cancer has been of consistent interest[19, 23, 24], patients value this form of data [25-28], and it underpins informed shared decision-making [29-32]. However, there is limited consensus on which areas of patient experience should be consistently assessed in brain tumour trials. In cancer trials using PROs, analyses are often unreported in publications and the clinical relevance of PRO results are overlooked [33]. A systematic review of glioma randomised controlled trials (RCTs) using PROs found that only 14% of these trials met the criteria for high quality reporting [34], with PRO results not being interpreted in 79%, and clinical relevance not discussed in 86% of trials.

There are international efforts to unify and improve practice. In PRO research in the field of neuro-oncology, the Response Assessment in Neuro-Oncology Patient Reported Outcomes (RANO-PRO) working group aims to provide guidance on Patient-Reported Outcome Measures (PROMs) in adult neuro-oncology clinical trials and practice (23). Their systematic review (26) found that 215 PROs have been used in brain tumour (primary and secondary) studies, the majority only used once or twice. The FDA and EMA recognise the importance of assessing symptoms, adverse effects and function as core constructs in all glioma trials[35], and have participated in an international multi-stakeholder workshop aiming to define a core set of priority constructs to be assessed as minimum in high grade glioma trials and care[36].

Core Outcome Sets (COS) establish ‘the minimum that should be measured and reported in all clinical trials of a specific condition’ [37], aiming to achieve consensus between researchers, clinicians, patients and policy makers. This facilitates consistent outcome collection, analysis, and reporting, enables data synthesis and meta-analyses, reduces research waste, and informs patient-centred care. Upon COS confirmation, further research will determine how to measure these outcomes.

The primary aim of this research is to finalise a COS for glioma comprising all outcome types, drawing on the UK perspective. We will define outcomes applicable to all glioma as well those that may be specific to glioma types. The COS will inform interpretation of the net clinical benefit of interventions in terms that reflect stakeholder priorities. Due to interest in core PROs in cancer, our secondary aim is to identify the COS outcomes which can be patient reported.

Aims and objectives

Aims: (1) to develop a COS from a UK perspective for use in adult primary glioma (astrocytoma, oligodendroglioma, oligoastrocytoma, ependymoma, astroblastoma, anaplastic ganglioglioma, glioblastoma, glioblastoma multiforme) phase III interventional trials; (2) identify COS outcomes which can be patient-reported.

For peer review only - http://bmjopen.bmj.com/site/about/guidelines.xhtml

Objectives:

1. Trial registry review to identify glioma trial outcomes and a systematic review of the qualitative literature to explore key stakeholders' research and treatment priorities;
2. Identify outcomes using qualitative interviews with glioma patients and caregivers;
3. Combine the results of Objectives 1 and 2 into a unified longlist of outcomes;
4. Achieve consensus on a COS through online Delphi process and a consensus meeting with a range of stakeholders.

Research questions:

- Which outcomes are important to patients, caregivers, and other key stakeholders, and what are the perceived gaps in current outcome assessment based on the lived experience of patients and caregivers?
- Can a COS be used across glioma trials or are specific subsets needed?
- Which of the identified outcomes can be patient-reported?
- How does the COS align with and inform the emerging international consensus on outcome assessment across brain tumour types?

Focus of COS

This COS will apply to phase III interventional trials for systemic anti-cancer treatments (including immunotherapy and chemotherapy), radiotherapy, surgery, and supportive care involving adults (aged over 18 years), diagnosed with glioma, with a specific focus on the UK population. Though some data formulating this COS will be drawn from a UK sample, trialists should consider its applicability internationally. To promote generalisability of results, recruitment into the qualitative interviews and Delphi exercise will be monitored for glioma type, age, ethnicity, and gender.

Methods

Study Design

COBra uses a mixed-methods, multi-stage approach in accordance with accepted COS methodology [38] and guidance[39] (Appendix 1) and registered with the Core Outcome Measures in Effectiveness Trials (COMET) Initiative[40].

Ethical Considerations

Ethical approval was granted (REF: SMREC 21/59). All data will be collected and stored in accordance with local regulations[41].

Study Team and Collaborators

The study team is multidisciplinary, including Patient and Public Involvement (PPI) representatives, healthcare professionals, researchers, policy makers, and regulators.

Collaboration between The Marie Curie Palliative Care Research Centre Cardiff (MCPCRC), the Centre for Patient Reported Outcomes Research (CPROR), the Centre for Trials Research (Cardiff University) and Birmingham Clinical Trials Unit (CTU) will underpin the methodological approach on behalf of the Supportive and Palliative Care subgroup of the

For peer review only - http://bmjopen.bmj.com/site/about/guidelines.xhtml

NCRI (National Cancer Research Institute) Brain Tumour group. Collaboration with the RANO-PRO Initiative[42] working group will ensure alignment with international efforts.

Patient and Public Involvement
The PPI team members contributed to study design and will develop and monitor the study as part of the Steering Group[43], contributing to data analysis and dissemination of study findings. The study team will seek advice from a wider panel of PPI representatives convened for the purpose of the study, consisting of individuals with a range of backgrounds and experiences. The detailed participation of PPI representatives will be reported in accordance with GRIPP2 [44].

Study Summary

There are five study stages:
Stage I: focusing on adult glioma, generate an outcome list from:
- (i) Registered glioma trials involving patients aged over 18 years and diagnosed with primary glioma;
- (ii) Qualitative literature exploring key stakeholder (including patients, caregivers, healthcare professionals, researchers, policymakers, regulators) research and treatment priorities.

Stage II: Semi-structured interviews with patients with primary glioma and their caregivers, exploring research and treatment priorities and generate an outcomes list.

Stage III: Review of Stage I and II outcome lists by the study team. Duplicates will be removed and the language will be checked for accessibility.

Stage IV: Delphi process with key stakeholder groups to rank the Stage III outcomes list. Thresholds for outcome inclusion in the COS will be determined *a priori*.

Stage V: A consensus meeting with key stakeholders to finalise a COS. Participants will review and discuss items in disagreement, agree the COS. If appropriate, specific subsets will be defined and agreed. The study team will identify which of the outcomes could be patient-reported.

Stage I – Evidence review
Aims
Review of clinical trial registries and a systematic review of published qualitative literature to generate an outcome list [38] from:

A) Phase III interventional glioma trials involving adult patients and diagnosed with primary glioma;
B) Qualitative studies exploring the lived experience and research priorities of adult patients with primary glioma, and other key stakeholders.

For peer review only - http://bmjopen.bmj.com/site/about/guidelines.xhtml

Search strategy and data extraction

Search A

ClinicalTrials.gov and ISRCTN clinical trials registries, based in the US and UK respectively, will be used to identify outcomes used in phase III interventional glioma trials in adults (Appendix 2). Data from both are available for public download. Where protocols are available alongside registration information, these will be retrieved.

Two reviewers will independently perform complete searches of glioma trials registered on clinicaltrials.gov and isrctn.com without restriction by date. The results will be independently reviewed for eligibility; disagreements will be resolved with a third reviewer. Two reviewers will independently extract data including basic trial information, year of study, primary outcome(s) and secondary outcomes. Data in the csv files will be cross-referenced with clinical trial registration entry for completeness, and with the protocol when available. The most recently updated of these will be used.

Trials sourced during Search A will be cross-referenced with those retrieved from the RANO-PRO study for information.

Search B

We will systematically review the qualitative literature describing the experiences and needs of adults diagnosed with glioma and thematically synthesise (44) their 'lived experiences' in relation to care, treatment and treatment outcomes.

Databases to be searched include MEDLINE, EMBASE, CINAHL, Web of Science, PsycINFO, the Cochrane Central Register of Controlled Trials and the Cochrane Library. Reference lists of key authors and journals will be hand searched. Qualitative studies, or mixed-method studies containing qualitative data, published in the English language, restricted to 15 years prior, will be included. Research involving adult patients and/or key stakeholders including informal carergivers, will be included. Two reviewers will independently review all titles and abstracts; a third reviewer will review citations for any disagreements. Full text studies will be reviewed by two reviewers; disagreements will be resolved with a third reviewer.

Two reviewers will independently extract data using a standardised data collection form, capturing the themes and sub-themes of the qualitative data pertaining to the lived experience of patients with primary glioma. The qualitative literature will be thematically synthesised following three stages: coding text, developing descriptive themes and generating themes[45]. The data will focus on patients and key stakeholders including informal carergivers, exploring their interpretation of patients' 'lived experiences', including views relating to their attitudes and experience of symptoms and functional outcomes. NVivo[46] will be used for data management.

Stage II – Interviews with patients and caregivers

Semi-structured interviews will be conducted with adults diagnosed with primary glioma across the spectrum of the disease. Interview participants can identify a caregiver to join them in an interview dyad. The interviews will inform the language used in the Delphi survey and identify outcomes not captured during Stage I.

For peer review only - http://bmjopen.bmj.com/site/about/guidelines.xhtml

Aims
The objectives of these interviews are to explore:
(i) outcomes that are important to patients;
(ii) caregivers' understanding of patients' priorities and experiences, as these may differ.

Participant eligibility and sampling
Dyads will comprise eligible patients histologically diagnosed with primary glioma (astrocytoma, oligodendroglioma, oligoastrocytoma, ependymoma, astroblastoma, anaplastic ganglioglioma, glioblastoma, glioblastoma multiforme) and a caregiver identified by the patient. Caregivers are defined as informal carers, who may be a family member or friend, who provides the majority of the support to the patient and is able to estimate the patient's priorities. Patients and caregivers will be over the age of 18 years.

Participants will be recruited through the NCRI Brain Group, the Tessa Jowell BRAIN MATRIX trial platform[47], CTUs, brainstrust – the brain cancer people, The Brain Tumour Charity, snowballing, known contacts, and social media platforms. Potential participants will be invited to contact the research team to express interest. Recruitment will be monitored to promote diversity in terms of glioma type, age, ethnicity, and gender, seeking balance between glioma types. Between 12 and 20 dyads representing the spectrum of malignant disease will be recruited based on previous studies and expected data saturation[48]. Data saturation will be assessed through constant discussion and evaluation of the data by the qualitative researchers conducting the data collection and analysis, together with members of the wider study team. Recruitment will end when data saturation is reached.

Consent and Capacity
Patients and caregivers will give consent on their own behalf if they wish to participate in an interview. If a patient or caregiver does not proceed with an interview, the other will still be invited to participate. Their permission is not required for the other to participate. Information sheets will be sent to eligible participants via post or email with the contact details of the research team member conducting the interviews. Participants expressing interest will be given the chance to ask any questions prior to consent. Participants will complete an electronic or hardcopy consent form or will be recorded giving verbal consent, depending on interview format.

In accordance with the Mental Capacity Act (2005), patient participants will be assumed to have capacity unless it is proven otherwise. If there is concern that the patient lacks capacity to participate, this will be discussed with the Chief Investigator, a clinician, about whether further research activity will occur. If research will not continue with the patient participant, the caregiver will be given the opportunity to take part in an interview to share their views.

Data collection
A semi-structured interview format will be used to understand patient experiences of living with glioma, and what they consider to be the most important outcomes from glioma treatment. Caregiver participants' perspective of patients' experience and priorities will be captured, not a direct report of the patients' condition. The interviews will be undertaken via phone or video link (e.g. Zoom or Microsoft Teams), or face-to-face, depending on the

For peer review only - http://bmjopen.bmj.com/site/about/guidelines.xhtml

situation and preference of patients. Interviews may take place with patients and caregivers together or separately, depending on their preference. Interviews where patients and caregivers are interviewed separately allow for differing views to be expressed. Where interviews are undertaken together, efforts will be made to ensure both are able to express their views. Interviews will be audio-recorded. The interview will be guided by open-ended questions on diagnosis, treatment, and their effects on patients and caregivers, directed towards understanding outcomes important to patients. The semi-structured format allows for spontaneous exploration of novel topics. The topic-guide may be reviewed and adapted iteratively after the first few interviews, if required. At the end of the interviews, participants will be asked directly which outcomes they believe should be measured in clinical trials. This places the lived experience of participants at the forefront, with patients and caregivers given the chance to talk about the things that matter most to them.

Data Analysis

The interview data, once transcribed and anonymised, will be thematically analysed [49] using NVivo software [46] for data management. Thematic analysis allows for the identification of patterns and themes within the data, to organise and describe data in rich detail[49]. It is particularly well-suited to studies that focus on lived experience. Data collected from patients and caregivers will be analysed and formulated into separate accounts.

Analysis of the first three transcripts will be conducted independently by two members of the research team experienced in qualitative research and a draft coding structure will be formulated. Disagreements in coding will be resolved through discussion and input from a third qualitative researcher will be sought when required. The draft coding frame will be reviewed by PPI team members and a coding structure for the remaining transcripts will be confirmed. The framework will be refined, until the analysis of all transcripts has been completed, with the findings synthesised into categories and subcategories.

Stage III – Review of outcome list

All outcomes, without limitation by outcome type, captured in Stage I will be grouped and classified [38]. Each grouping will contain domains and subdomains that broadly measure particular aspects of the effects of interventions (e.g. symptoms and function)[50]. The outcome lists formed by each of the two researchers will be compared for completeness, and differences in the categorisation will be resolved through discussion.

The categories and subcategories generated in Stage II will be formulated into an outcome list and differences in the categorisation will be resolved through discussion.

A longlist of outcomes will be generated from the Stage I and II outcome lists. Duplicates will be removed during this process. This list will be reviewed by the study team to refine the language used to describe the outcomes. The team will review the structure of the questions included in the Delphi survey. At this stage, it will be decided whether separate Delphi processes are needed according to glioma type based on the emerging data.

Stage IV – Delphi survey

For peer review only - http://bmjopen.bmj.com/site/about/guidelines.xhtml

For peer review only

A modified two-round Delphi will be used to assess the relative importance of outcomes included in the stage III outcome list. Participants will be invited to consider applicability of the COS to new and emerging therapies, and whether the outcomes would apply. The aim of the Delphi process is to reach consensus on which outcomes should form the COS for glioma trials.

Recruitment

Approximately 100 participants with professional or personal experience of glioma care and treatment: 1) patients, 2) caregivers, 3) healthcare professionals and researchers, 4) policy-makers and regulators will be recruited as previously described in earlier stages. During Delphi registration participants will choose the stakeholder group with which they most identify but can note if they identify with other stakeholder groups besides their primary. Approximately 25 participants will be recruited to each stakeholder group, recruitment will be monitored and will inform and direct efforts as required. Consent will be taken electronically during the online registration process.

Delphi process

The Delphi exercise will reflect COMET recommendations [38] and will present the Stage III outcome list. Participants will rate each of the outcomes on a 9-point Likert scale, (1–3, not important; 4–6, important but not critical; and 7–9, important and critical)[51]. During Round 1, participants can add outcomes they feel are missing. Votes from individuals in each stakeholder group will be given equal weighting. All original outcomes will be presented in Round 2. Outcomes added by participants in Round 1 will be presented in Round 2. In Round 2, respondents will be presented with their own rating for each outcome and how it was rated by their own stakeholder group. Based on this information, respondents will be invited to amend their score, if they wish. During Round 2, participants can rate the outcomes suggested in Round 1.

The threshold for consensus for inclusion in or exclusion from the COS will be ≥70%, informed by those used in comparable COS development studies [52, 53]. After the Delphi, outcomes will be proposed for inclusion in the final COS if ≥70% respondents rate the item as 7-9 and ≤15% rate the item as 1-3. Items will be proposed for exclusion from the final COS if ≥70% respondents rate the item as 1-3 and ≤15% rate the item as 7-9. Those outcomes that do not reach agreement after the two Delphi rounds will be discussed in the consensus meeting, together with the items proposed for inclusion and exclusion.

Missing data

To minimise partial response, participants will be unable to skip questions but can indicate when they feel unable to rank specific items. Reminders will be used to minimise participant attrition between Delphi rounds. Use of specialised Delphi software, Delphi Manager, will enable rapid inter-round rating calculations to allow the second round to open with minimal delay to further reduce attrition.

Stage V consensus meeting

This meeting may be held virtually or in person, depending on the situation and preference of the majority of participants. All Delphi participants will be invited. Notes will be taken

For peer review only - http://bmjopen.bmj.com/site/about/guidelines.xhtml

during the meeting and consent will be sought from all participants to audio-record the meeting for reference. Decisions made during the consensus meeting will be made through anonymous voting using voting software. Decisions will proceed if ratified by ≥70% of the group. In cases where there is <100% consensus, decisions will be discussed until those in disagreement are satisfied that their views have been considered and that the decision can proceed. The core outcomes applicable to all glioma trials will be agreed, as will any outcomes identified as specific to particular types of glioma. Following the consensus meeting, the study team will identify which of the outcomes could be assessed by patient reporting.

Dissemination

The final COS will be published in compliance with accepted reporting standards [38] and adopted and promoted by the NCRI Brain Clinical Studies Group Supportive and Palliative Care subgroup for use in glioma studies. The subgroup will publish a position statement mandating for UK CTUs involved in brain tumour research to implement the COS.

Study findings will be disseminated widely, including to national and international conferences and high-impact journals. A plain English summary will be co-produced with PPI team members and made available to participants upon request. The COS will be promoted amongst patient and carer groups using The Brain Tumour Charity network (including BRIAN), NCRI and regional PPI frameworks, brainstrust, and other patient organisations. The importance of COS development is increasingly recognised by funders, such as the National Institute for Health Research, and regulators, such as EMA and FDA. The COS will therefore be promoted to encourage its inclusion in 'justification of outcomes' sections of funding proposals and regulatory submissions. The final COS will be freely available on the COMET database.

Though some data used to develop this COS will be drawn from a UK perspective, its applicability internationally should be explored. The study team will consider the findings of this study in the context of existing international initiatives. Findings will be shared with international partners and may be integrated into international guidance on outcome assessment across all brain tumour types.

COBra will directly collaborate with the RANO-PRO working group and affiliated international initiatives to share the UK perspective. Following study completion, RANO-PRO findings may be used to select appropriate COAs aligned to the COS. COBra will also collaborate with UK funders, trialists and CTUs on COS implementation and the consistent application of international standards for collection, analysis and reporting of the COS across all UK studies.

Funding

This study is funded by The Brain Tumour Charity (GN-000704). The funders have no role in study design or manuscript preparation.

Competing interests

Due to their involvement in the study design, the study team members will not participate in the Delphi process or consensus meeting, other than in a facilitative role. Study team members will

For peer review only - http://bmjopen.bmj.com/site/about/guidelines.xhtml

encourage engagement and participation in the Delphi process and consensus meeting by individuals within the networks of which they are part, as appropriate.

Author contribution

The study concept and design was conceived by AR, SS, HS, AN, HB, KS, RG, RA, CW, OLA, PK, SCR, LD, EB, MC, and AB. MC and AR advised on methodology. EB and AR will undertake the registry review, EB and SS will undertake the qualitative systematic review. EB and SS will recruit, screen and consent participants and will undertake the interviews with input from AR and AB. EB will recruit for the Delphi and consensus meeting, with input from SS, AR, and AB. HS prepared the first draft of the manuscript. AR prepared subsequent drafts. SS, HS, AN, HB, KS, RG, RA, CW, OLA, PK, SCR, LD, EB, MC, and AB all provided edits and critiqued the manuscript for intellectual content.

References

1. Diseases, N.O.f.R. *Glioma*. 2019 [cited 2021 21 September].
2. Goodenberger, M.L. and R.B. Jenkins, *Genetics of adult glioma.* Cancer Genet, 2012. **205**(12): p. 613-21.
3. Wood, M.D., A.M. Halfpenny, and S.R. Moore, *Applications of molecular neuro-oncology - a review of diffuse glioma integrated diagnosis and emerging molecular entities.* Diagnostic Pathology, 2019. **14**(1): p. 29.
4. UK, C.R. *Survival*. 2019 [cited 2021 1 June].
5. Sagberg, L.M., O. Solheim, and A.S. Jakola, *Quality of survival the 1st year with glioblastoma: a longitudinal study of patient-reported quality of life*. Journal of Neurosurgery JNS, 2016. **124**(4): p. 989-997.
6. Basch, E., et al., *Patient-Reported Outcomes in Cancer Drug Development and US Regulatory Review: Perspectives From Industry, the Food and Drug Administration, and the Patient.* JAMA Oncol, 2015. **1**(3): p. 375-9.
7. MacDonald, L.o.b.o.t.N.-O.G. *Top 10 priorities for clinical research in primary brain and spinal cord tumours: final report of the James Lind Alliance Priority Setting Partnership in Neuro-Oncology.* 2015 28 January 2021].
8. Collins, A., et al., *The challenges and suffering of caring for people with primary malignant glioma: qualitative perspectives on improving current supportive and palliative care practices.* BMJ Support Palliat Care, 2014. **4**(1): p. 68-76.
9. Piil, K., et al., *Controlled rehabilitative and supportive care intervention trials in patients with high-grade gliomas and their caregivers: a systematic review.* BMJ Support Palliat Care, 2016. **6**(1): p. 27-34.
10. Armstrong, T.S. and M.R. Gilbert, *Net clinical benefit: functional endpoints in brain tumor clinical trials.* Curr Oncol Rep, 2007. **9**(1): p. 60-5.
11. Administration, U.F.a.D. *Clinical Outcome Assessment (COA) Qualification Program*. 2021 [cited 2021 1 June].
12. FDA. *Guidance for industry: Patient-Reported outcome measures: Use in medical product development to support labeling claims*. 2009 [cited 2021 2 June].
13. Fairclough, D.L., *Patient reported outcomes as endpoints in medical research.* Statistical Methods in Medical Research, 2004. **13**(2): p. 115-138.

For peer review only - http://bmjopen.bmj.com/site/about/guidelines.xhtml

14. Hirsch, B.R., et al., *Characteristics of oncology clinical trials: insights from a systematic analysis of ClinicalTrials.gov.* JAMA Intern Med, 2013. **173**(11): p. 972-9.
15. Dirven, L., et al., *Systematic review on the use of patient-reported outcome measures in brain tumor studies: part of the Response Assessment in Neuro-Oncology Patient-Reported Outcome (RANO-PRO) initiative.* Neuro-Oncology Practice, 2021.
16. Kirkham, J.J., et al., *Can a core outcome set improve the quality of systematic reviews?--a survey of the Co-ordinating Editors of Cochrane Review Groups.* Trials, 2013. **14**: p. 21.
17. Administration, F.a.D. *FDA Patient-Focused Drug Development Guidance Series for Enhancing the Incorporation of the Patient's Voice in Medical Product Development and Regulatory Decision Making*. 2020 [cited 2021 3 August].
18. *Guidance for industry: patient-reported outcome measures: use in medical product development to support labeling claims: draft guidance.* Health Qual Life Outcomes, 2006. **4**: p. 79.
19. Administration, F.a.D. *Core Patient-Reported Outcomes in Cancer Clinical Trials: Draft Guidance for Industry*. 2021 [cited 2021 3 August].
20. Agency, E.M. *Appendix 2 to the guideline on the evaluation of anticancer medicinal products in man : The use of patient-reported outcome (PRO) measures in oncology studies*. 2016 [cited 2021 3 August].
21. Basch, E. and C. Yap, *Patient-Reported Outcomes for Tolerability Assessment in Phase I Cancer Clinical Trials.* JNCI: Journal of the National Cancer Institute, 2021. **113**(8): p. 943-944.
22. Kluetz, P.G., et al., *Informing the Tolerability of Cancer Treatments Using Patient-Reported Outcome Measures: Summary of an FDA and Critical Path Institute Workshop.* Value Health, 2018. **21**(6): p. 742-747.
23. Reeve, B.B., et al., *Recommended Patient-Reported Core Set of Symptoms to Measure in Adult Cancer Treatment Trials.* JNCI: Journal of the National Cancer Institute, 2014. **106**(7).
24. Kluetz, P.G., et al., *Focusing on Core Patient-Reported Outcomes in Cancer Clinical Trials: Symptomatic Adverse Events, Physical Function, and Disease-Related Symptoms.* Clin Cancer Res, 2016. **22**(7): p. 1553-8.
25. Calvert, M.J. and N. Freemantle, *Use of health-related quality of life in prescribing research. Part 1: why evaluate health-related quality of life?* J Clin Pharm Ther, 2003. **28**(6): p. 513-21.
26. Ouwens, M., et al., *Development of indicators for patient-centred cancer care.* Support Care Cancer, 2010. **18**(1): p. 121-30.
27. Brundage, M., et al., *Cancer patients' preferences for communicating clinical trial quality of life information: a qualitative study.* Qual Life Res, 2003. **12**(4): p. 395-404.
28. Davison, B.J., et al., *Provision of individualized information to men and their partners to facilitate treatment decision making in prostate cancer.* Oncol Nurs Forum, 2003. **30**(1): p. 107-14.
29. England, N. *Shared decision making*. 2021 [cited 2021 1 June].
30. Excellence, N.I.f.H.a.C. *Shared decision making*. 2021 [cited 2021 1 June].
31. Au, H.J., et al., *Added value of health-related quality of life measurement in cancer clinical trials: the experience of the NCIC CTG.* Expert Rev Pharmacoecon Outcomes Res, 2010. **10**(2): p. 119-28.
32. Basch, E., *Toward patient-centered drug development in oncology.* N Engl J Med, 2013. **369**(5): p. 397-400.
33. Pe, M., et al., *Statistical analysis of patient-reported outcome data in randomised controlled trials of locally advanced and metastatic breast cancer: a systematic review.* Lancet Oncol, 2018. **19**(9): p. e459-e469.
34. Dirven, L., et al., *The level of patient-reported outcome reporting in randomised controlled trials of brain tumour patients: a systematic review.* Eur J Cancer, 2014. **50**(14): p. 2432-48.

For peer review only - http://bmjopen.bmj.com/site/about/guidelines.xhtml

35. Armstrong, T.S., et al., *Glioma patient-reported outcome assessment in clinical care and research: a Response Assessment in Neuro-Oncology collaborative report.* The Lancet Oncology, 2020. **21**(2): p. e97-e103.
36. Helfer, J.L., et al., *Report of the Jumpstarting Brain Tumor Drug Development Coalition and FDA clinical trials clinical outcome assessment endpoints workshop (October 15, 2014, Bethesda MD).* Neuro-oncology, 2016. **18 Suppl 2**(Suppl 2): p. ii26-ii36.
37. Kirkham, J.J., et al., *Core Outcome Set-STAndards for Development: The COS-STAD recommendations.* PLoS Med, 2017. **14**(11): p. e1002447.
38. Williamson, P.R., et al., *The COMET Handbook: version 1.0.* Trials, 2017. **18**(Suppl 3): p. 280.
39. Kirkham, J.J., et al., *Core Outcome Set-STAndardised Protocol Items: the COS-STAP Statement.* Trials, 2019. **20**(1): p. 116.
40. Byrne A, S.S., Scott H, Nelson A, Seddon K, Calvert M, Retzer A, Grant R, Bulbeck H, Adams R, Watts C, Aiyegbusi OL, Kearns P, Cruz Rivera S. *Patient Reported Core Outcomes in Brain Tumour Trials: the COBra study*. 2020 [cited 2021 5 August].
41. Department for Digital, C., Media & Sport, Home Office. *Data Protection Act 2018*. 2018 [cited 2021 1 February].
42. Dirven, L., et al., *Working plan for the use of patient-reported outcome measures in adults with brain tumours: a Response Assessment in Neuro-Oncology (RANO) initiative.* Lancet Oncol, 2018. **19**(3): p. e173-e180.
43. INVOLVE. *UK Standards for Public Involvement: Better public involvement for better health and social care*. 2019 [cited 2021 4 August].
44. Staniszewska, S., et al., *GRIPP2 reporting checklists: tools to improve reporting of patient and public involvement in research.* Bmj, 2017. **358**: p. j3453.
45. Thomas, J. and A. Harden, *Methods for the thematic synthesis of qualitative research in systematic reviews.* BMC Med Res Methodol, 2008. **8**: p. 45.
46. Ltd, Q.I.P. *NVivo qualitative data analysis software*. 2020 [cited 2021 1 June].
47. Birmingham, U.o. *Tessa Jowell BRAIN MATRIX*. 2020 [cited 2021 5 August].
48. Saunders, B., et al., *Saturation in qualitative research: exploring its conceptualization and operationalization.* Qual Quant, 2018. **52**(4): p. 1893-1907.
49. Braun, V. and V. Clarke, *Using thematic analysis in psychology.* Qualitative Research in Psychology, 2006. **3**(2): p. 77-101.
50. Health, N.I.o. *Patient-Reported Outcomes Measurement Information System–PROMIS*. 2019 [cited 2021 29 January].
51. Guyatt, G.H., et al., *GRADE guidelines: 2. Framing the question and deciding on important outcomes.* J Clin Epidemiol, 2011. **64**(4): p. 395-400.
52. Waters, A.M., et al., *The CONSENSUS study: protocol for a mixed methods study to establish which outcomes should be included in a core outcome set for oropharyngeal cancer.* Trials, 2014. **15**: p. 168.
53. Fish, R., et al., *Core outcome research measures in anal cancer (CORMAC): protocol for systematic review, qualitative interviews and Delphi survey to develop a core outcome set in anal cancer.* BMJ Open, 2017. **7**(11): p. e018726.

For peer review only - http://bmjopen.bmj.com/site/about/guidelines.xhtml

Appendix 1

		Page
Title 1a	Identify in the title that the paper describes the protocol for the planned development of a COS	1
Abstract 1b	Provide a structured abstract	2
INTRODUCTION Background and objectives 2a	Describe the background and explain the rationale for developing the COS, and identify the reasons why a COS is needed and the potential barriers to its implementation	3,4
2b	Describe the specific objectives with reference to developing a COS	5
Scope 3a	Describe the health condition(s) and population(s) that will be covered by the COS	5
3b	Describe the intervention(s) that will be covered by the COS	5
3c	Describe the context of use for which the COS is to be applied	5
METHODS Stakeholders 4	Describe the stakeholder groups to be involved in the COS development process, the nature of and rationale for their involvement and also how the individuals will be identified; this should cover involvement both as members of the research team and as participants in the study	6-10
Information sources 5a	Describe the information sources that will be used to identify the list of outcomes. Outline the methods or reference other protocols/papers	6-8
5b	Describe how outcomes may be dropped/ combined, with reasons	9-11
Consensus process 6	Describe the plans for how the consensus process will be undertaken	10-11
Consensus definition 7a	Describe the consensus definition	10-11
7b	Describe the procedure for determining how outcomes will be added/combined/dropped from consideration during the consensus process	10-11
ANALYSIS Outcome scoring/ feedback 8	Describe how outcomes will be scored and summarised, describe how participants will receive feedback during the consensus process	10-11
Missing data 9	Describe how missing data will be handled during the consensus process	10
ETHICS and DISSEMINATION Ethics approval/ informed consent 10	Describe any plans for obtaining research ethics committee/institutional review board approval in relation to the consensus process and describe how informed consent will be obtained (if relevant)	5, 8, 10
Dissemination 11	Describe any plans to communicate the results to study participants and COS users, inclusive of methods and timing of dissemination	11
ADMINISTRATIVE INFORMATION Funders 12	Describe sources of funding, role of funders	11
Conflicts of interest 13	Describe any potential conflicts of interest within the study team and how they will be managed	11

For peer review only

For peer review only

Appendix 2

1. ClinicalTrials.gov
Condition/Disease: glioma OR astrocytoma OR oligodendroglioma OR oligoastrocytoma OR ependymoma OR astroblastoma OR anaplastic gangioglioma OR glioblastoma OR GBM OR Glioblastoma multiforme
Study type: Interventional Studies (Clinical Trials)
Age: Adult 18-64 AND Older Adult (65+)
Phase: III

2. ISRCTN.com
Each term searched individually:
Condition/Disease: glioma; astrocytoma; oligodendroglioma; oligoastrocytoma; ependymoma; astroblastoma; anaplastic gangioglioma; glioblastoma; GBM; Glioblastoma multiforme

For peer review only

BMJ Open

Development of a Core Outcome Set and Identification of Patient-Reportable Outcomes for Primary Brain Tumour Trials: Protocol for the COBra Study

Journal:	*BMJ Open*
Manuscript ID	bmjopen-2021-057712.R1
Article Type:	Protocol
Date Submitted by the Author:	11-May-2022
Complete List of Authors:	Retzer, Ameeta; University of Birmingham, Centre for Patient Reported Outcomes Research, Institute for Applied Health Research; National Institute for Health and Care Research (NIHR), Applied Research Centre, West Midlands<br>Sivell, Stephanie; Cardiff University, Marie Curie Palliative Care Research Centre, Division of Population Medicine, Cardiff University School of Medicine, College of Biomedical and Life Sciences<br>Scott, Hannah; University of Cambridge, Cambridge Public Health, University of Cambridge School of Clinical Medicine<br>Nelson, Annmarie; Cardiff University School of Medicine, Marie Curie Palliative Care Research Centre, Division of Population Medicine, Cardiff University School of Medicine, College of Biomedical and Life Sciences<br>Bulbeck, Helen; brainstrust<br>Seddon, Kathy; Cardiff University<br>Grant, Robin ; Department of Clinical Neurosciences, Royal Infirmary of Edinburgh<br>Adams, Richard; University of Cardiff, Centre for Trials Research<br>Watts, Colin; University of Birmingham, Institute of Cancer and Genomic Sciences, College of Medical and Dental Sciences<br>Aiyegbusi, Olalekan Lee; University of Birmingham, Centre for Patient Reported Outcomes Research, Institute of Applied Health Research; University of Birmingham, NIHR Birmingham Biomedical Research Centre, University of Birmingham and University Hospitals Birmingham NHS Foundation Trust<br>Kearns, Pamela; University of Birmingham, Institute of Cancer and Genomic Sciences, College of Medical and Dental Sciences; University of Birmingham, NIHR Birmingham Biomedical Research Centre<br>Cruz Rivera, Samantha; University of Birmingham, Centre for Patient Reported Outcomes Research, Institute of Applied Health Research; University of Birmingham, Birmingham Health Partners Centre for Regulatory Science and Innovation<br>Dirven, Linda; Leiden University, Department of Neurology, Leiden University Medical Center; Medical Centre Haaglanden, Department of Neurology<br>Baddeley, Elin; Cardiff University, Marie Curie Palliative Care Research Centre, Division of Population Medicine, Cardiff University School of Medicine, College of Biomedical and Life Sciences<br>Calvert , Melanie ; University of Birmingham, Centre for Patient Reported Outcomes Research, Institute of Applied Health Research; National

	Institute for Health and Care Research (NIHR), Applied Research Centre West Midlands<br>Byrne, Anthony; Cardiff University, Marie Curie Palliative Care Research Centre, Division of Population Medicine, Cardiff University School of Medicine, College of Biomedical and Life Sciences
**Primary Subject Heading**:	Oncology
Secondary Subject Heading:	Palliative care, Patient-centred medicine, Qualitative research, Research methods
Keywords:	Neurological oncology < NEUROLOGY, Neurological oncology < ONCOLOGY, Adult palliative care < PALLIATIVE CARE, QUALITATIVE RESEARCH, Clinical trials < THERAPEUTICS

SCHOLARONE™ Manuscripts

Development of a Core Outcome Set and Identification of Patient-Reportable Outcomes for Primary Brain Tumour Trials: Protocol for the COBra Study

Authors: Ameeta Retzer[1,2,3], Stephanie Sivell[4], Hannah Scott[5], Annmarie Nelson[4], Helen Bulbeck[6], Kathy Seddon[7], Robin Grant[8], Richard Adams[9], Colin Watts[10], Olalekan Lee Aiyegbusi[1,2,3,11,12], Pamela Kearns[10,12, 13], Samantha Cruz Rivera[1,2,11], Linda Dirven[14,15], Elin Baddeley[4], Melanie Calvert[1,2,3,11,12,16, 17] , Anthony Byrne[4]

[1]Institute of Applied Health Research, University of Birmingham, Birmingham, UK

[2]Centre for Patient Reported Outcomes Research, Institute of Applied Health Research, University of Birmingham, Birmingham, UK

[3]National Institute for Health and Care Research (NIHR) Applied Research Centre West Midlands, Birmingham, UK

[4] Marie Curie Palliative Care Research Centre, Division of Population Medicine, Cardiff University School of Medicine, College of Biomedical and Life Sciences, UK

[5] Cambridge Public Health, University of Cambridge School of Clinical Medicine, UK

[6] Brainstrust - the brain cancer people, UK

[7]Cardiff University, UK

[8] Department of Clinical Neurosciences, Royal Infirmary of Edinburgh, UK

[9] Centre for Trials Research, Cardiff University, UK

[10] Institute of Cancer and Genomic Sciences, College of Medical and Dental Sciences, University of Birmingham, UK

[11]Birmingham Health Partners Centre for Regulatory Science and Innovation, University of Birmingham, Birmingham, UK

[12]NIHR Birmingham Biomedical Research Centre, University of Birmingham, Birmingham, UK

[13] Cancer Research UK Clinical Trials Unit (CRCTU), Institute of Cancer and Genomic Sciences, University of Birmingham, UK[14] Department of Neurology, Leiden University Medical Center, Leiden, The Netherlands

[15] Department of Neurology, Haaglanden Medical Center, The Hague, The Netherlands

For peer review only - http://bmjopen.bmj.com/site/about/guidelines.xhtml

[16]NIHR Surgical Reconstruction and Microbiology Research Centre, University of Birmingham, Birmingham, UK

[17]Midlands Health Data Research UK, Birmingham, UK

Correspondence to: Professor Anthony Byrne (anthony.byrne2@wales.nhs.uk)

Abstract:

Introduction
Primary brain tumours, specifically gliomas, are a rare disease group. The disease and treatment negatively impacts on patients and those close to them. The high rates of physical and cognitive morbidity differ from other cancers causing reduced health-related quality of life. Glioma trials using outcomes that allow holistic analysis of treatment benefits and risks enable informed care decisions. Currently, outcome assessment in glioma trials is inconsistent, hindering evidence synthesis. A core outcome set (COS): an agreed minimum set of outcomes to be measured and reported may address this. International initiatives focus on defining core outcomes assessments across brain tumour types. This protocol describes the development of a COS for use in glioma trials, applicable across glioma types involving UK stakeholders, with provision to identify subsets as required. Due to stakeholder interest in data reported from the patient perspective, outcomes from the COS that can be patient-reported will be identified.
Methods and analysis
Stage I: (i) trial registry review to identify outcomes collected in glioma trials and (ii) systematic review of qualitative literature exploring glioma patient and key stakeholder research priorities. Stage II: semi-structured interviews with glioma patients and caregivers. Outcome lists will be generated from Stages I and II. Stage III: study team will remove duplicate items from the outcome lists and ensure accessible terminology for inclusion in the Delphi survey. Stage IV: a two-round Delphi process whereby the outcomes will be rated by key stakeholders. Stage V: a consensus meeting where participants will finalise the COS. The study team will identify the COS outcomes that can be patient-reported. Further research is needed to match patient-reported outcomes to available measures.
Ethics and dissemination
Ethical approval was obtained (REF SMREC 21/59, Cardiff University School of Medicine Research Ethics Committee). Study findings will be disseminated widely through conferences and journal publication. The final COS will be adopted and promoted by patient and carer groups and its use by funders encouraged.
Trial and PROSPERO registration
Core Outcome Measures in Effectiveness Trials (https://www.comet-initiative.org/Studies/Details/1793); PROSPERO (CRD42021236979).

For peer review only - http://bmjopen.bmj.com/site/about/guidelines.xhtml

For peer review only

Strengths and Limitations

- This study collects original qualitative data to ensure all outcomes prioritised by glioma patients are identified. However, this is a resource-intensive process that may not be available to all core outcome set developers.
- Review of trial registries represents a pragmatic approach to comprehensively identify outcomes used in trials rather than reliance on often incomplete outcome reporting in glioma trial publications[1]. There are limitations to this approach - use of trial registries means those that are not registered will not be identified, registry use is inconsistent globally, completeness and specificity can be questionable, and updating of entries continues to be a challenge[2]. However, the quality of registration has been observed to be improving and trial registration associated with subsequent publication and use of the same outcomes as defined in their protocols as in their published reports[3].
- Bias may be introduced by inviting qualitative interview participants from Stage II to take part in Delphi; though this encourages familiarity with concepts, enabling meaningful participation in the Delphi
- Qualitative data collected from the UK population may limit international applicability, though this allows exploration of issues that may be specific to UK context and validation of this COS for use in other settings should be explored.

Introduction:

Primary brain tumours, specifically gliomas, are part of a rare disease group[4]. The disease and its treatment have negative effects on patients and those close to them. The high rates of physical and cognitive morbidity differ from other cancers, with significant impact on a wide range of functional domains. Gliomas are the commonest form of primary brain tumour[5], accounting for 80% of malignant brain tumours. Gliomas represent a heterogeneous group of cancers with variable outcome, traditionally graded from I to IV (least to most aggressive) [2]. However, rapid developments in molecular diagnostics have led to refinements in nomenclature, suggesting a more nuanced approach to brain tumours classification[6]. This would acknowledge the spectrum ranging from a variable but slower-progressing course, such as oligodendroglioma or astrocytoma, to fast-growing tumours such as glioblastoma, a particularly aggressive subtype with a median survival of 12 to 15 months and 5% five year survival rate[7].

The poor prognosis of some glioma patients and the high symptom burden has led to a growing emphasis on their quality of survival[8]. Maintaining cognitive function, physical function and other health-related quality of life aspects throughout the disease trajectory are key considerations alongside very modest survival benefits captured through traditional metrics of tumour response and overall or progression-free survival, particularly for patients with aggressive forms of glioma [9]. Therefore, it is important that glioma intervention studies collect a range of data aligned with patient priorities to enable assessment of the net clinical benefit of treatments [10-13].

Data collected to evidence effects of interventions are known as “outcomes”. Outcomes include traditional measures such as progression-free survival and radiological tumour response but also Clinical Outcome Assessments (COAs). COAs describe how a patient feels,

For peer review only - http://bmjopen.bmj.com/site/about/guidelines.xhtml

functions, or survives. COAs include Clinician Reported Outcomes (ClinRO), Observer Reported Outcomes (ObsRO), Performance Outcomes (PerfO), and Patient Reported Outcomes (PROs)[14]. PROs assess a range of outcomes including symptoms, functional health, well-being and psychological issues from the patients' perspective, without interpretation by a clinician or anyone else [15]. When assessing treatments, PROs enable insight into the impact of treatment on patient's perceived wellbeing where other outcome data that may indicate minimal differences in disease control and survival, potentially influencing patients' treatment choices [16].

Interpreting the clinical benefit of treatments requires effective data synthesis and meta-analyses of trial outcomes. This requires consistent use of outcomes, use of appropriate outcome measures, and diligent data capture, analysis and reporting. Inconsistent outcome use is widespread. A significant lack of standard ontology has been found in cancer clinical trials [17] and in brain tumour studies specifically[18]. Moreover, selective outcome and missing data reporting is common[19], introducing bias and hindering evidence synthesis. PROs are critical to the comprehensive evaluation of treatment benefits and side effects, and are increasingly used by regulatory authorities. The Food and Drug Administration (FDA) is prioritising a patient-centred approach to drug development[20], a consistent approach to PRO use generally [21], and in cancer clinical trials specifically[22].The European Medicines Agency (EMA) support PRO use to assess drug efficacy and tolerability in informing product approval in cancer[23], consistent with the FDA[24, 25]. Key PROs for use in cancer has been of consistent interest[22, 26, 27], patients value this form of data [28-31], and it underpins informed shared decision-making [32-35]. However, there is limited consensus on which areas of patient experience should be consistently assessed in brain tumour trials. In cancer trials using PROs, analyses are often unreported in publications and the clinical relevance of PRO results are overlooked [36]. A systematic review of glioma randomised controlled trials (RCTs) using PROs found that only 14% of these trials met the criteria for high quality reporting [37], with PRO results not being interpreted in 79%, and clinical relevance not discussed in 86% of trials.

There are international efforts to unify and improve practice. In PRO research in the field of neuro-oncology, the Response Assessment in Neuro-Oncology Patient Reported Outcomes (RANO-PRO) working group aims to provide guidance on Patient-Reported Outcome Measures (PROMs) in adult neuro-oncology clinical trials and practice (23). Their systematic review (26) found that 215 PROs have been used in brain tumour (primary and secondary) studies, the majority only used once or twice. The FDA and EMA recognise the importance of assessing symptoms, adverse effects and function as core constructs in all glioma trials[38], and have participated in an international multi-stakeholder workshop aiming to define a core set of priority constructs to be assessed as minimum in high grade glioma trials and care[39].

Core Outcome Sets (COS) establish 'the minimum that should be measured and reported in all clinical trials of a specific condition' [40], aiming to achieve consensus between researchers, clinicians, patients and policy makers. This facilitates consistent outcome collection, analysis, and reporting, enables data synthesis and meta-analyses, reduces research waste, and informs patient-centred care. Upon COS confirmation, further research will determine how to measure these outcomes.

For peer review only - http://bmjopen.bmj.com/site/about/guidelines.xhtml

The primary aim of this research is to develop a COS from a UK perspective for use in adult primary glioma (astrocytoma, oligodendroglioma, oligoastrocytoma, ependymoma, astroblastoma, anaplastic ganglioglioma, glioblastoma, glioblastoma multiforme) phase III interventional trials comprising all outcome types. We will define outcomes applicable to all glioma as well those that may be specific to glioma types. The COS will inform interpretation of the net clinical benefit of interventions in terms that reflect stakeholder priorities. Due to interest in core PROs in cancer, our secondary aim is to identify the COS outcomes which can be patient reported.

Focus of COS
This COS will apply to phase III interventional trials for systemic anti-cancer treatments (including immunotherapy and chemotherapy), radiotherapy, surgery, and supportive care involving adults (aged over 18 years), diagnosed with glioma, with a specific focus on the UK population. Though some data formulating this COS will be drawn from a UK sample, trialists should consider the COS to be applicable internationally. To promote generalisability of results, recruitment into the qualitative interviews and Delphi exercise will be monitored for glioma type, age, ethnicity, and gender.

Methods and analysis

Objectives:

1. Trial registry review to identify glioma trial outcomes and a systematic review of the qualitative literature to explore key stakeholders' research and treatment priorities;
2. Identify outcomes using qualitative interviews with glioma patients and caregivers;
3. Combine the results of Objectives 1 and 2 into a unified longlist of outcomes;
4. Achieve consensus on a COS through online Delphi process and a consensus meeting with a range of stakeholders.

Study Design
The COBra (Patient Reported Core Outcomes in Brain Tumour Trials) study uses a mixed-methods, multi-stage approach in accordance with accepted COS methodology [41] and guidance[42] (Appendix 1) and registered with the Core Outcome Measures in Effectiveness Trials (COMET) Initiative[43].

Study Team and Collaborators
The study team is multidisciplinary, including Patient and Public Involvement (PPI) representatives, healthcare professionals, researchers, policy makers, and regulators.

The Marie Curie Palliative Care Research Centre Cardiff, the Centre for Patient Reported Outcomes Research, the Centre for Trials Research (Cardiff University) and Birmingham

For peer review only - http://bmjopen.bmj.com/site/about/guidelines.xhtml

Clinical Trials Unit (CTU) will provide methodological steer on behalf of the Supportive and Palliative Care subgroup of the NCRI (National Cancer Research Institute) Brain Tumour group. Collaboration with the RANO-PRO Initiative[44] working group will ensure alignment with international efforts.

Patient and Public Involvement

The PPI team members contributed to study design and will develop and monitor the study as part of the Steering Group[45], contributing to data analysis and dissemination of study findings. The study team will seek advice from a wider panel of PPI representatives convened for the purpose of the study, consisting of individuals with a range of backgrounds and experiences. The detailed participation of PPI representatives will be reported in accordance with GRIPP2 (Guidance for Reporting Involvement of Patients and the Public) [46].

Stage I – Evidence review

Aims

Review of clinical trial registries and a systematic review of published qualitative literature to generate an outcome list [41] from:

A) Phase III interventional glioma trials involving adult patients and diagnosed with primary glioma;
B) Qualitative studies exploring the lived experience and research priorities of adult patients with primary glioma, and other key stakeholders.

Search strategy and data extraction

Search A

ClinicalTrials.gov and ISRCTN clinical trials registries, based in the US and UK respectively, will be used to identify outcomes used in phase III interventional glioma trials in adults (Appendix 2). Data from both are available for public download. Where protocols are available alongside registration information, these will be retrieved.

Two reviewers will independently perform complete searches of glioma trials registered on clinicaltrials.gov and isrctn.com without restriction by date. The results will be independently reviewed for eligibility; disagreements will be resolved with a third reviewer. Two reviewers will independently extract data including basic trial information, year of study, primary outcome(s) and secondary outcomes. Data in the csv files will be cross-referenced with clinical trial registration entry for completeness, and with the protocol when available. The most recently updated of these will be used.

Trials sourced during Search A will be cross-referenced with those retrieved from the RANO-PRO study for information.

Search B

For peer review only - http://bmjopen.bmj.com/site/about/guidelines.xhtml

We will systematically review the qualitative literature describing the experiences and needs of adults diagnosed with glioma and thematically synthesise (44) their 'lived experiences' in relation to care, treatment and treatment outcomes.

Databases to be searched include MEDLINE, EMBASE, CINAHL, Web of Science, PsycINFO, the Cochrane Central Register of Controlled Trials and the Cochrane Library. Reference lists of key authors and journals will be hand searched. Qualitative studies, or mixed-method studies containing qualitative data, published in the English language, restricted to 15 years prior, will be included. This is because of limited data prior and literature captured is more reflective of current treatment options and patient perspective Research involving adult patients and/or key stakeholders including informal carergivers, will be included. Two reviewers will independently review all titles and abstracts; a third reviewer will review citations for any disagreements. Full text studies will be reviewed by two reviewers; disagreements will be resolved with a third reviewer.

Two reviewers will independently extract data using a standardised data collection form, capturing the themes and sub-themes of the qualitative data pertaining to the lived experience of patients with primary glioma. The qualitative literature will be thematically synthesised following three stages: coding text, developing descriptive themes and generating themes[47]. The data will focus on patients and key stakeholders including informal carergivers, exploring their interpretation of patients' 'lived experiences', including views relating to their attitudes and experience of symptoms and functional outcomes. NVivo[48] will be used for data management.

Stage II – Interviews with patients and caregivers

Semi-structured interviews will be conducted with adults diagnosed with primary glioma across the spectrum of the disease. Interview participants can identify a caregiver to join them in an interview dyad. The interviews will inform the language used in the Delphi survey and identify outcomes not captured during Stage I.

Aims

The objectives of these interviews are to explore:

(i) outcomes that are important to patients;
(ii) caregivers' understanding of patients' priorities and experiences, as these may differ.

Participant eligibility and sampling

Dyads will comprise eligible patients histologically diagnosed with primary glioma (astrocytoma, oligodendroglioma, oligoastrocytoma, ependymoma, astroblastoma, anaplastic ganglioglioma, glioblastoma, glioblastoma multiforme) and a caregiver identified by the patient. Caregivers are defined as informal carers, who may be a family member or friend, who provides the majority of the support to the patient and is able to estimate the patient's priorities. Patients and caregivers will be over the age of 18 years.

Participants will be recruited through the NCRI Brain Group, the Tessa Jowell BRAIN MATRIX trial platform[49], CTUs, brainstrust – the brain cancer people, The Brain Tumour Charity, snowballing, known contacts, and social media platforms. Potential participants will be

For peer review only - http://bmjopen.bmj.com/site/about/guidelines.xhtml

invited to contact the research team to express interest. Recruitment will be monitored to promote diversity in terms of glioma type, age, ethnicity, and gender, seeking balance between glioma types. Between 12 and 20 dyads representing the spectrum of malignant disease will be recruited based on previous studies and expected data saturation[50]. Data saturation will be assessed through constant discussion and evaluation of the data by the qualitative researchers conducting the data collection and analysis, together with members of the wider study team. Recruitment will end when data saturation is reached.

Consent and Capacity

Patients and caregivers will give consent on their own behalf if they wish to participate in an interview. If a patient or caregiver does not proceed with an interview, the other will still be invited to participate. Their permission is not required for the other to participate. Information sheets will be sent to eligible participants via post or email with the contact details of the research team member conducting the interviews. Participants expressing interest will be given the chance to ask any questions prior to consent. Participants will complete an electronic or hardcopy consent form or will be recorded giving verbal consent, depending on interview format.

In accordance with the Mental Capacity Act (2005), patient participants will be assumed to have capacity unless it is proven otherwise. If there is concern that the patient lacks capacity to participate, this will be discussed with the Chief Investigator, a clinician, about whether further research activity will occur. If research will not continue with the patient participant, the caregiver will be given the opportunity to take part in an interview to share their views.

Data collection

A semi-structured interview format will be used to understand patient experiences of living with glioma, and what they consider to be the most important outcomes from glioma treatment. Caregiver participants' perspective of patients' experience and priorities will be captured, not a direct report of the patients' condition. The interviews will be undertaken via phone or video link (e.g. Zoom or Microsoft Teams), or face-to-face, depending on the situation and preference of patients. Interviews may take place with patients and caregivers together or separately, depending on their preference. Interviews where patients and caregivers are interviewed separately allow for differing views to be expressed. Where interviews are undertaken together, efforts will be made to ensure both are able to express their views. Interviews will be audio-recorded. The interview will be guided by open-ended questions on diagnosis, treatment, and their effects on patients and caregivers, directed towards understanding outcomes important to patients. The semi-structured format allows for spontaneous exploration of novel topics. The topic-guide may be reviewed and adapted iteratively after the first few interviews, if required. At the end of the interviews, participants will be asked directly which outcomes they believe should be measured in clinical trials. This places the lived experience of participants at the forefront, with patients and caregivers given the chance to talk about the things that matter most to them.

Data Analysis

The interview data, once transcribed and anonymised, will be thematically analysed [51] using NVivo software [48] for data management. A preliminary framework will be derived from the available literature including the Thematic analysis allows for the identification of

For peer review only - http://bmjopen.bmj.com/site/about/guidelines.xhtml

patterns and themes within the data, to organise and describe data in rich detail[51]. It is particularly well-suited to studies that focus on lived experience. Data collected from patients and caregivers will be analysed and formulated into separate accounts.

Analysis of the first three transcripts will be conducted independently by two members of the research team experienced in qualitative research and a draft coding structure will be formulated. Disagreements in coding will be resolved through discussion and input from a third qualitative researcher will be sought when required. The draft coding frame will be reviewed by PPI team members and a coding structure for the remaining transcripts will be confirmed. The framework will be refined, until the analysis of all transcripts has been completed, with the findings synthesised into categories and subcategories.

Stage III – Review of outcome list

All outcomes, without limitation by outcome type, captured in Stage I will be grouped and classified [41]. A broad ontology for this will be developed from the framework outlined in the COMET handbook and relevant frameworks from the available literature[38] in advance of outcome extraction and will be iteratively refined based on the outcomes identified. The ontology will serve as a categorical tool to organise and present the outcomes in an accessible manner. Each grouping will contain domains and subdomains that broadly measure particular aspects of the effects of interventions (e.g. symptoms and function)[52]. The outcome lists formed by each of the two researchers will be compared for completeness, and differences in the categorisation will be resolved through discussion.

The categories and subcategories generated in Stage II will be formulated into an outcome list and differences in the categorisation will be resolved through discussion.

A longlist of outcomes will be generated from the Stage I and II outcome lists. Duplicates will be removed during this process. This list will be reviewed by the study team to refine the language used to describe the outcomes. The team will review the structure of the questions included in the Delphi survey. At this stage, it will be decided whether separate Delphi processes are needed according to glioma type based on the emerging data.

Stage IV – Delphi survey

A modified two-round Delphi will be used to assess the relative importance of outcomes included in the stage III outcome list. Participants will be invited to consider applicability of the COS to new and emerging therapies, and whether the outcomes would apply. The aim of the Delphi process is to reach consensus on which outcomes should form the COS for glioma trials.

Recruitment

Approximately 100 participants with professional or personal experience of glioma care and treatment: 1) patients, 2) caregivers, 3) healthcare professionals and researchers, 4) policy-makers and regulators will be recruited as previously described in earlier stages. During Delphi registration participants will choose the stakeholder group with which they most identify but can note if they identify with other stakeholder groups besides their primary. Approximately 25 participants will be recruited to each stakeholder group, recruitment will

For peer review only - http://bmjopen.bmj.com/site/about/guidelines.xhtml

be monitored and will inform and direct efforts as required. Consent will be taken electronically during the online registration process.

Delphi process

The Delphi exercise will reflect COMET recommendations [41] and will present the Stage III outcome list. Participants will rate each of the outcomes on a 9-point Likert scale, (1–3, not important; 4–6, important but not critical; and 7–9, important and critical)[53]. During Round 1, participants can add outcomes they feel are missing. Votes from individuals in each stakeholder group will be given equal weighting. All original outcomes will be presented in Round 2. Outcomes added by participants in Round 1 will be presented in Round 2. In Round 2, respondents will be presented with their own rating for each outcome and how it was rated by their own stakeholder group. Based on this information, respondents will be invited to amend their score, if they wish. During Round 2, participants can rate the outcomes suggested in Round 1.

The threshold for consensus for inclusion in or exclusion from the COS will be ≥70%, informed by those used in comparable COS development studies [54, 55]. After the Delphi, outcomes will be proposed for inclusion in the final COS if ≥70% respondents rate the item as 7-9 and ≤15% rate the item as 1-3. Items will be proposed for exclusion from the final COS if ≥70% respondents rate the item as 1-3 and ≤15% rate the item as 7-9. Those outcomes that do not reach agreement after the two Delphi rounds will be discussed in the consensus meeting, together with the items proposed for inclusion and exclusion.

Missing data

To minimise partial response, participants will be unable to skip questions but can indicate when they feel unable to rank specific items. Reminders will be used to minimise participant attrition between Delphi rounds. Use of specialised Delphi software, Delphi Manager, will enable rapid inter-round rating calculations to allow the second round to open with minimal delay to further reduce attrition.

Stage V consensus meeting

This meeting may be held virtually or in person, depending on the situation and preference of the majority of participants. All Delphi participants will be invited. Notes will be taken during the meeting and consent will be sought from all participants to audio-record the meeting for reference. Decisions made during the consensus meeting will be made through anonymous voting using voting software. Decisions will proceed if ratified by ≥70% of the group. In cases where there is <100% consensus, decisions will be discussed until those in disagreement are satisfied that their views have been considered and that the decision can proceed. This meeting allows for a further opportunity to discuss, validate and the confirm the final COS. The core outcomes applicable to all glioma trials will be agreed, as will any outcomes identified as specific to particular types of glioma. Following the consensus meeting, the study team will identify which of the outcomes could be assessed by patient reporting.

Ethics and dissemination

For peer review only - http://bmjopen.bmj.com/site/about/guidelines.xhtml

Ethical approval was granted (REF: SMREC 21/59, Cardiff University School of Medicine Research Ethics Committee). All data will be collected and stored in accordance with local regulations[56].

The final COS will be published in compliance with accepted reporting standards [41] and adopted and promoted by the NCRI Brain Clinical Studies Group Supportive and Palliative Care subgroup for use in glioma studies. The subgroup will publish a position statement mandating for UK CTUs involved in brain tumour research to implement the COS.
Study findings will be disseminated widely, including to national and international conferences and high-impact journals. A plain English summary will be co-produced with PPI team members and made available to participants upon request. The COS will be promoted amongst patient and carer groups using The Brain Tumour Charity network (including BRIAN), NCRI and regional PPI frameworks, brainstrust, and other patient organisations. The importance of COS development is increasingly recognised by funders, such as the National Institute for Health and Care Research, and regulators, such as EMA and FDA. The COS will therefore be promoted to encourage its inclusion in 'justification of outcomes' sections of funding proposals and regulatory submissions. The final COS will be freely available on the COMET database.

Though some data used to develop this COS will be drawn from a UK perspective, the trial registry searches were without restriction based on country and the qualitative literature were limited to those in English language only. The study steering committee has membership stakeholders leading international initiatives and the Delphi survey and consensus meeting will involve participants from international regulatory bodies. As a result, the resulting COS should be considered to be internationally applicable. For use in other settings or countries, validation exercises are advised to ensure economic and cultural differences are integrated. The study team will consider the findings of this study in the context of existing international initiatives. Findings will be shared with international partners and may be integrated into international guidance on outcome assessment across all brain tumour types.
COBra will directly collaborate with the RANO-PRO working group and affiliated international initiatives to share the UK perspective. Following study completion, RANO-PRO findings may be used to select appropriate COAs aligned to the COS. COBra will also collaborate with UK funders, trialists and CTUs on COS implementation and the consistent application of international standards for collection, analysis and reporting of the COS across all UK studies.

Following finalising the COS, further research is required to identify and/or develop corresponding outcome measures.

References

For peer review only - http://bmjopen.bmj.com/site/about/guidelines.xhtml

1. Tardy, M.P., et al., *Quality of Randomized Controlled Trials Reporting in the Treatment of Adult Patients with High-Grade Gliomas.* Oncologist, 2018. **23**(3): p. 337-345.
2. Viergever, R.F. and K. Li, *Trends in global clinical trial registration: an analysis of numbers of registered clinical trials in different parts of the world from 2004 to 2013.* BMJ Open, 2015. **5**(9): p. e008932.
3. Chan, A.-W., et al., *Association of Trial Registration With Reporting of Primary Outcomes in Protocols and Publications.* JAMA, 2017. **318**(17): p. 1709-1711.
4. Diseases, N.O.f.R. *Glioma*. 2019 [cited 2021 21 September].
5. Goodenberger, M.L. and R.B. Jenkins, *Genetics of adult glioma.* Cancer Genet, 2012. **205**(12): p. 613-21.
6. Wood, M.D., A.M. Halfpenny, and S.R. Moore, *Applications of molecular neuro-oncology - a review of diffuse glioma integrated diagnosis and emerging molecular entities.* Diagnostic Pathology, 2019. **14**(1): p. 29.
7. UK, C.R. *Survival*. 2019 [cited 2021 1 June].
8. Sagberg, L.M., O. Solheim, and A.S. Jakola, *Quality of survival the 1st year with glioblastoma: a longitudinal study of patient-reported quality of life.* Journal of Neurosurgery JNS, 2016. **124**(4): p. 989-997.
9. Basch, E., et al., *Patient-Reported Outcomes in Cancer Drug Development and US Regulatory Review: Perspectives From Industry, the Food and Drug Administration, and the Patient.* JAMA Oncol, 2015. **1**(3): p. 375-9.
10. MacDonald, L.o.b.o.t.N.-O.G. *Top 10 priorities for clinical research in primary brain and spinal cord tumours: final report of the James Lind Alliance Priority Setting Partnership in Neuro-Oncology.* 2015 28 January 2021].
11. Collins, A., et al., *The challenges and suffering of caring for people with primary malignant glioma: qualitative perspectives on improving current supportive and palliative care practices.* BMJ Support Palliat Care, 2014. **4**(1): p. 68-76.
12. Piil, K., et al., *Controlled rehabilitative and supportive care intervention trials in patients with high-grade gliomas and their caregivers: a systematic review.* BMJ Support Palliat Care, 2016. **6**(1): p. 27-34.
13. Armstrong, T.S. and M.R. Gilbert, *Net clinical benefit: functional endpoints in brain tumor clinical trials.* Curr Oncol Rep, 2007. **9**(1): p. 60-5.
14. Administration, U.F.a.D. *Clinical Outcome Assessment (COA) Qualification Program*. 2021 [cited 2021 1 June].
15. FDA. *Guidance for industry: Patient-Reported outcome measures: Use in medical product development to support labeling claims*. 2009 [cited 2021 2 June].
16. Fairclough, D.L., *Patient reported outcomes as endpoints in medical research.* Statistical Methods in Medical Research, 2004. **13**(2): p. 115-138.
17. Hirsch, B.R., et al., *Characteristics of oncology clinical trials: insights from a systematic analysis of ClinicalTrials.gov.* JAMA Intern Med, 2013. **173**(11): p. 972-9.
18. Dirven, L., et al., *Systematic review on the use of patient-reported outcome measures in brain tumor studies: part of the Response Assessment in Neuro-Oncology Patient-Reported Outcome (RANO-PRO) initiative.* Neuro-Oncology Practice, 2021.
19. Kirkham, J.J., et al., *Can a core outcome set improve the quality of systematic reviews?--a survey of the Co-ordinating Editors of Cochrane Review Groups.* Trials, 2013. **14**: p. 21.
20. Administration, F.a.D. *FDA Patient-Focused Drug Development Guidance Series for Enhancing the Incorporation of the Patient's Voice in Medical Product Development and Regulatory Decision Making*. 2020 [cited 2021 3 August].
21. *Guidance for industry: patient-reported outcome measures: use in medical product development to support labeling claims: draft guidance.* Health Qual Life Outcomes, 2006. **4**: p. 79.

For peer review only - http://bmjopen.bmj.com/site/about/guidelines.xhtml

22. Administration, F.a.D. *Core Patient-Reported Outcomes in Cancer Clinical Trials: Draft Guidance for Industry*. 2021 [cited 2021 3 August].
23. Agency, E.M. *Appendix 2 to the guideline on the evaluation of anticancer medicinal products in man : The use of patient-reported outcome (PRO) measures in oncology studies*. 2016 [cited 2021 3 August].
24. Basch, E. and C. Yap, *Patient-Reported Outcomes for Tolerability Assessment in Phase I Cancer Clinical Trials.* JNCI: Journal of the National Cancer Institute, 2021. **113**(8): p. 943-944.
25. Kluetz, P.G., et al., *Informing the Tolerability of Cancer Treatments Using Patient-Reported Outcome Measures: Summary of an FDA and Critical Path Institute Workshop.* Value Health, 2018. **21**(6): p. 742-747.
26. Reeve, B.B., et al., *Recommended Patient-Reported Core Set of Symptoms to Measure in Adult Cancer Treatment Trials.* JNCI: Journal of the National Cancer Institute, 2014. **106**(7).
27. Kluetz, P.G., et al., *Focusing on Core Patient-Reported Outcomes in Cancer Clinical Trials: Symptomatic Adverse Events, Physical Function, and Disease-Related Symptoms.* Clin Cancer Res, 2016. **22**(7): p. 1553-8.
28. Calvert, M.J. and N. Freemantle, *Use of health-related quality of life in prescribing research. Part 1: why evaluate health-related quality of life?* J Clin Pharm Ther, 2003. **28**(6): p. 513-21.
29. Ouwens, M., et al., *Development of indicators for patient-centred cancer care.* Support Care Cancer, 2010. **18**(1): p. 121-30.
30. Brundage, M., et al., *Cancer patients' preferences for communicating clinical trial quality of life information: a qualitative study.* Qual Life Res, 2003. **12**(4): p. 395-404.
31. Davison, B.J., et al., *Provision of individualized information to men and their partners to facilitate treatment decision making in prostate cancer.* Oncol Nurs Forum, 2003. **30**(1): p. 107-14.
32. England, N. *Shared decision making*. 2021 [cited 2021 1 June].
33. Excellence, N.I.f.H.a.C. *Shared decision making*. 2021 [cited 2021 1 June].
34. Au, H.J., et al., *Added value of health-related quality of life measurement in cancer clinical trials: the experience of the NCIC CTG.* Expert Rev Pharmacoecon Outcomes Res, 2010. **10**(2): p. 119-28.
35. Basch, E., *Toward patient-centered drug development in oncology.* N Engl J Med, 2013. **369**(5): p. 397-400.
36. Pe, M., et al., *Statistical analysis of patient-reported outcome data in randomised controlled trials of locally advanced and metastatic breast cancer: a systematic review.* Lancet Oncol, 2018. **19**(9): p. e459-e469.
37. Dirven, L., et al., *The level of patient-reported outcome reporting in randomised controlled trials of brain tumour patients: a systematic review.* Eur J Cancer, 2014. **50**(14): p. 2432-48.
38. Armstrong, T.S., et al., *Glioma patient-reported outcome assessment in clinical care and research: a Response Assessment in Neuro-Oncology collaborative report.* The Lancet Oncology, 2020. **21**(2): p. e97-e103.
39. Helfer, J.L., et al., *Report of the Jumpstarting Brain Tumor Drug Development Coalition and FDA clinical trials clinical outcome assessment endpoints workshop (October 15, 2014, Bethesda MD).* Neuro-oncology, 2016. **18 Suppl 2**(Suppl 2): p. ii26-ii36.
40. Kirkham, J.J., et al., *Core Outcome Set-STAndards for Development: The COS-STAD recommendations.* PLoS Med, 2017. **14**(11): p. e1002447.
41. Williamson, P.R., et al., *The COMET Handbook: version 1.0.* Trials, 2017. **18**(Suppl 3): p. 280.
42. Kirkham, J.J., et al., *Core Outcome Set-STAndardised Protocol Items: the COS-STAP Statement.* Trials, 2019. **20**(1): p. 116.
43. Byrne A, S.S., Scott H, Nelson A, Seddon K, Calvert M, Retzer A, Grant R, Bulbeck H, Adams R, Watts C, Aiyegbusi OL, Kearns P, Cruz Rivera S. *Patient Reported Core Outcomes in Brain Tumour Trials: the COBra study*. 2020 [cited 2021 5 August].

For peer review only - http://bmjopen.bmj.com/site/about/guidelines.xhtml

44. Dirven, L., et al., *Working plan for the use of patient-reported outcome measures in adults with brain tumours: a Response Assessment in Neuro-Oncology (RANO) initiative.* Lancet Oncol, 2018. **19**(3): p. e173-e180.
45. INVOLVE. *UK Standards for Public Involvement: Better public involvement for better health and social care*. 2019 [cited 2021 4 August].
46. Staniszewska, S., et al., *GRIPP2 reporting checklists: tools to improve reporting of patient and public involvement in research.* Bmj, 2017. **358**: p. j3453.
47. Thomas, J. and A. Harden, *Methods for the thematic synthesis of qualitative research in systematic reviews.* BMC Med Res Methodol, 2008. **8**: p. 45.
48. Ltd, Q.I.P. *NVivo qualitative data analysis software*. 2020 [cited 2021 1 June].
49. Birmingham, U.o. *Tessa Jowell BRAIN MATRIX*. 2020 [cited 2021 5 August].
50. Saunders, B., et al., *Saturation in qualitative research: exploring its conceptualization and operationalization.* Qual Quant, 2018. **52**(4): p. 1893-1907.
51. Braun, V. and V. Clarke, *Using thematic analysis in psychology.* Qualitative Research in Psychology, 2006. **3**(2): p. 77-101.
52. Health, N.I.o. *Patient-Reported Outcomes Measurement Information System–PROMIS*. 2019 [cited 2021 29 January].
53. Guyatt, G.H., et al., *GRADE guidelines: 2. Framing the question and deciding on important outcomes.* J Clin Epidemiol, 2011. **64**(4): p. 395-400.
54. Waters, A.M., et al., *The CONSENSUS study: protocol for a mixed methods study to establish which outcomes should be included in a core outcome set for oropharyngeal cancer.* Trials, 2014. **15**: p. 168.
55. Fish, R., et al., *Core outcome research measures in anal cancer (CORMAC): protocol for systematic review, qualitative interviews and Delphi survey to develop a core outcome set in anal cancer.* BMJ Open, 2017. **7**(11): p. e018726.
56. Department for Digital, C., Media & Sport, Home Office. *Data Protection Act 2018*. 2018 [cited 2021 1 February].

Author contribution

The study concept and design was conceived by AR, SS, HS, AN, HB, KS, RG, RA, CW, OLA, PK, SCR, LD, EB, MC, and AB. MC and AR advised on methodology. EB and AR will undertake the registry review, EB and SS will undertake the qualitative systematic review. EB and SS will recruit, screen and consent participants and will undertake the interviews with input from AR and AB. EB will recruit for the Delphi and consensus meeting, with input from SS, AR, and AB. HS prepared the first draft of the manuscript. AR prepared subsequent drafts. SS, HS, AN, HB, KS, RG, RA, CW, OLA, PK, SCR, LD, EB, MC, and AB all provided edits and critiqued the manuscript for intellectual content.

Funding statement

This study is funded by The Brain Tumour Charity (GN-000704). The funders have no role in study design or manuscript preparation.

Competing interests

Due to their involvement in the study design, the study team members will not participate in the Delphi process or consensus meeting, other than in a facilitative role. Study team members will encourage engagement and participation in the Delphi process and consensus meeting by individuals within the networks of which they are part, as appropriate.

For peer review only

Appendix 1

		Page
Title 1a	Identify in the title that the paper describes the protocol for the planned development of a COS	1
Abstract 1b	Provide a structured abstract	2
INTRODUCTION Background and objectives 2a	Describe the background and explain the rationale for developing the COS, and identify the reasons why a COS is needed and the potential barriers to its implementation	3-5, 11
2b	Describe the specific objectives with reference to developing a COS	5
Scope 3a	Describe the health condition(s) and population(s) that will be covered by the COS	5
3b	Describe the intervention(s) that will be covered by the COS	5
3c	Describe the context of use for which the COS is to be applied	5
METHODS Stakeholders 4	Describe the stakeholder groups to be involved in the COS development process, the nature of and rationale for their involvement and also how the individuals will be identified; this should cover involvement both as members of the research team and as participants in the study	7-10
Information sources 5a	Describe the information sources that will be used to identify the list of outcomes. Outline the methods or reference other protocols/papers	6-10
5b	Describe how outcomes may be dropped/ combined, with reasons	9-10
Consensus process 6	Describe the plans for how the consensus process will be undertaken	10
Consensus definition 7a	Describe the consensus definition	10
7b	Describe the procedure for determining how outcomes will be added/combined/dropped from consideration during the consensus process	10
ANALYSIS Outcome scoring/ feedback 8	Describe how outcomes will be scored and summarised, describe how participants will receive feedback during the consensus process	10
Missing data 9	Describe how missing data will be handled during the consensus process	10
ETHICS and DISSEMINATION Ethics approval/ informed consent 10	Describe any plans for obtaining research ethics committee/institutional review board approval in relation to the consensus process and describe how informed consent will be obtained (if relevant)	9-11
Dissemination 11	Describe any plans to communicate the results to study participants and COS users, inclusive of methods and timing of dissemination	11
ADMINISTRATIVE INFORMATION Funders 12	Describe sources of funding, role of funders	14
Conflicts of interest 13	Describe any potential conflicts of interest within the study team and how they will be managed	14

For peer review only

For peer review only

Appendix 2

1. ClinicalTrials.gov
Condition/Disease: glioma OR astrocytoma OR oligodendroglioma OR oligoastrocytoma OR ependymoma OR astroblastoma OR anaplastic gangioglioma OR glioblastoma OR GBM OR Glioblastoma multiforme
Study type: Interventional Studies (Clinical Trials)
Age: Adult 18-64 AND Older Adult (65+)
Phase: III

2. ISRCTN.com
Each term searched individually:
Condition/Disease: glioma; astrocytoma; oligodendroglioma; oligoastrocytoma; ependymoma; astroblastoma; anaplastic gangioglioma; glioblastoma; GBM; Glioblastoma multiforme

For peer review only

Comment	Author response
**Editor**<br><br>Please include the name of the ethics committee that approved your study in the Ethics and Dissemination section of the Abstract. Please also include a dissemination statement in this section.	Thank you, this has now been added.
Please also include the name of the ethics committee that approved this study in the ethics statement in the main text.	Thank you, this has now been added.
Please reformat the main text so that it follows the structure recommended in the journal's instructions for authors for study protocols, for example the main text of your manuscript should contain an Ethics and Dissemination section. See: https://bmjopen.bmj.com/pages/authors/#protocol	Thank you, the titles have been updated and the ethics statement is now included in the Ethics and Dissemination section.
Along with your revised manuscript, please include a copy of the COS-STAP checklist indicating the page/line numbers of your manuscript where the relevant information can be found (https://doi.org/10.1186/s13063-019-3230-x)	Thank you – this has now been updated and attached following the edits resulting from the editorial and reviewer comments.
**Additional changes**	Minor edits have been made to update affiliations.
**Reviewer 1**<br><br>Glioma outcome trials should have perspective from all the stakeholders . a comprehensive outcome measure would give a better choice of selection and planning for all concerned .<br>this kind of study to measure outcome in a comprehensive way is welcome .	Thank you for your support of this work. Rather than the development or recommendation of an outcome measurement instrument such as a specific questionnaire, this work aims to identify and finalise the core outcomes to be collected and reported in glioma interventional trials, which can be subsequently aligned with measurement tool(kit)s/instruments.
I suggest the survey questions should be comprehensive and should not be just question number 123 etc but stakeholder should be able to answer q no 8 .<br>there should be provision for missing data which the participant can answer separately .	We recognise that currently used questionnaires / instruments may be burdensome for participants, and mechanisms to address this and minimise missing data are essential. With the development of a core outcome set (COS), the aim is to represent the *minimum* required outcomes to be collected and reported in glioma studies. This will promote consistency in outcome use rather than volume of data, and may reduce missing data.<br>For the Delphi survey to be conducted in our project, we hope to get input from the participants on *all* the proposed items, as these are deemed relevant in stages I-III of

For peer review only

	the project. Of course participants have the option to provide explanations on their choice, including an explanation when they choose not to rate a specific item.
many of the questionares fall short on this when the paticipant does not attempt an answer or the answer is not any of the options .<br>this can be corrected if apilot study is conducted and the lacunae of the survey proproforma is correted .	Thank you for this comment. In this project we will ensure that the perspective of all stakeholders, including patients, is represented in our work in all stages of the work. Indeed, patients and carers will be included in qualitative interviews, and all stakeholders will take part in the Delphi survey and consensus meeting. In this project, we will provide recommendations on the core outcomes that should be measured, not the specific instruments. The choice for an appropriate instrument should be based on relevance (content validity), but also on other psychometric properties of the instrument, as well as patient burden. This work will be done in the future – this has now been stated in the ethics and dissemination section.
also i think the consensus statment should be different for different regions keeping in mind economic cultural differences	We agree that it is important to consider cross cultural differences. The current COS will be developed from a UK perspective only and aims to include participants from the diverse UK population (i.e. heterogeneous population, reflective of the UK population). For use in other settings or countries, we advise validation exercises to ensure economic and cultural differences are integrated. Wording relating to this has been added to the ethics and dissemination section.
Reviewer 2<br><br>1. Scope of COS - the team discuss international applicability, but also confirm that the data used to develop the COS will be primarily UK-based. Have the authors considered international recruitment to the patient interviews, Delphi survey and consensus meeting?	Thank you for this comment. Due to the specific experiences and priorities of UK patients within the UK health system, we limited our recruitment to the UK. However, there will be some international participation in the Delphi survey through representation from international regulatory stakeholders. Also, international collaborators are represented on our steering committee and through engagement with colleagues from the Medicines and Healthcare products Regulatory Agency (MHRA), we will be advised on international alignment. Nevertheless, for the COS to be used in other countries / cultures, further validation is necessary. Wording to detail

For peer review only

	this has been added to the Ethics and Dissemination section.
2. Identifying potentially important outcomes.<br>a) Could the authors expand on their justification for using protocols and trial registry entries to identify potentially important outcomes, rather than the widely adopted systematic review of published scientific literature? What would the limitations of this be given that, in my experience from similar studies, trial registries can be notoriously inaccurate and sparse with respect to details on which outcomes are planned to be reported. Whilst there is not a single 'best' way to do this, the team's approach should have its strengths and weaknesses discussed.	We agree that this is important to discuss and further justification for rationale for using trial registries has therefore been added to the limitations section of the manuscript.
b) What time period will be covered for the trial registries and qualitative literature review and what is the justification for this?	No limitation was placed on the trial registry search and a 15 year limitation was placed on the qualitative literature search. The rationale for this limit was due to the limited data prior to this point and that the literature captured is more reflective of current treatment options and patient perspective. Wording relating to this has been added to the text.
c) The qualitative literature review is an interesting approach to identifying potentially important outcomes. If there is sufficient body of work in this field, what is the need to undertake a further set of interviews which are resource intensive and costly? As things stand, COS are financially costly and take a long time to develop. This approach will be a valuable methodological consideration for future COS developers.	Thank you for this insightful observation. This strategy was carefully considered by the steering committee and the study management group. We felt secondary analyses of qualitative literature is limited by the primary interpretation of the data. Including interviews in our project allows in depth exploration of the patient's perspective and analytical clarity on how this may be reflected in outcomes across the spectrum of glioma. We acknowledge that this is an important question and it is a resource intensive process and not necessarily appropriate for use in all COS. Use of qualitative interviews is encouraged in the COMET (Core Outcome Measures in Effectiveness Trials) handbook and their use was carefully considered in this study. A note relating to this has been added to the strengths and limitations section. We will report on the number of unique outcomes identified from this source to add to evidence base and inform future COS.
d) If interviews are necessary, how many are planned, or predicted to be necessary?	Between 12 and 20 dyads representing the spectrum of malignant disease will be recruited based on previous studies and

For peer review only

	expected data saturation. Data saturation will be assessed through constant discussion and evaluation of the data by the qualitative researchers conducting the data collection and analysis, together with members of the wider study team. Recruitment will end when data saturation is reached. This is currently reported in the methods section.
e) How will the longlist of outcomes be rationalised into items presented in the Delphi survey? What framework will be used and how many items does the team envisage is an ideal number for participants to prioritise?	Details relating to the development of the outcomes lists are now more extensively reported in stage III of the methods, and is in accordance with the approach outlined in the COMET handbook.
Reviewer 3<br><br>(1) Introduction, page 3, second para: The sentence "These data are known as..." does not follow from the previous statement. Outcomes are any effects of interventions, not just patient priorities, or clinical benefits. It also includes adverse events and many more .	Following the reviewer's suggestion, this sentence has been re-phrased and linked with the next paragraph.
(2) Introduction, page 3, fourth para: Please delete "net benefit", it is about effects or effectiveness in general.	The word "net" has been removed.
(3) Introduction, page 4, fourth para: What do you mean by "finalise"? Reading the entire protocol, it seems like a new and independent project. If work hast already been done, please describe this transparently.	This was indeed not phrased clearly. We have now clarified that this is a new independent project where a COS will be developed and finalised in a consensus meeting.
(4) Introduction, page 4, fourth para: The aim of COS is to make trial results comparable worldwide. If a COS is going to be developed, it must be done using an international perspective. Focusing on a specific country perspective makes no sense, because we don't want to have country specific COS. The statement in the Dissemination part "... applicability internationally should be explored..." (page 11) is also very weak. What does "The study team will consider the findings of .... Existing international initiatives..." mean? If there are international initiatives, they must be involved.	Thank you for this feedback. There are three sources of data used in the identification of outcomes – the trial registries, qualitative literature, and qualitative interviews. The trial registry searches were without restriction based on country and the qualitative literature was limited to those in English language only. Both of these can be considered as international sources. However, the qualitative interviews were only undertaken in the UK so their experiences and priorities may be specific to and shaped by the UK health system. The study steering committee has membership stakeholders leading international initiatives and the Delphi survey and consensus meeting will involve participants from international regulatory bodies. The statement in the introduction describing the UK perspective has been removed and

For peer review only

	the statement in the dissemination section has been re-phrased. This has also been re-worded in the "Focus of COS" section. Nevertheless, for the COS to be used in other countries / cultures, further validation is necessary. Wording relating to this has now been added to the ethics and dissemination section.
(5) Introduction, aim, methods: There is a lot of repetition regarding aims and objectives. The objectives are described in the Background (COS-STAP item 2b) and they don't need to be repeated again and again. The "Objectives" (page 5) belong to the methods and the "Research questions" seems to be little bit out of scope. Especially the last bullet is strange (see above). Later in the methods (page 6) the aims are stated again. List the aims once, the present the methods accordingly.	The aims and objectives section has been split out so the aims are included in the background section and the repetitive parts are removed. The objectives have been moved to the methods section. The research questions have been removed. We hope that the adjusted manuscript reads better.
(6) Methods, study design, page 5: Please name COBra first, before using the acronym.	Thank you for noticing – this has now been corrected.
(7) Methods, team members, page 5: Please just list who is doing what. Sentences such as "…. underpin the methodological approach…" are strange. Please check whether all abbreviations (MCPCRC etc.) are actually needed later in the text. Please explain what PPI and GRIPP is (page 6).	Thank you – this has been re-worded to clarify the role. The abbreviations that are not used again in the manuscript have been removed and GRIPP2 has been provided in full. PPI is given in full in the first section of the study team section.
(8) Methods, study summary, page 6: Please delete.	Following the reviewer's suggestion, this has been deleted.
(9) Methods, search A, page 7: Please consider to look at published trials too.	Given the under-reporting of outcomes in trial publications, we decided to use trial registries instead. This issue was also raised by reviewer 1 and further information for the rationale and exploration of the limitations of this approach have been added to the manuscript.
(10) Methods, stage III, page 9: How exactly will the extracted outcomes classified? My recommendation is to look what was reported and then develop/define the domains inductively and present these outcomes as they are. Classification may be done later.	The approach for this has been provided in more detail, outlining the development of an ontology and its use as a categorical tool.
(11) Delphi study: I would recommend to decide later, whether two rounds are sufficient. It really depends on the length of the list and the voting results. If too many outcome are considered critical, then another voting is necessary.	Thank you for raising this issue, which we have carefully discussed when setting up the study. The decision to proceed with two rounds was determined in advance as the details of participation are required for informed consent of participants. Two rounds were decided to mitigate attrition between rounds and minimise missing data, reduce time required of participants and promote data completeness. Further

For peer review only

	to this, the study team was conscious of participant burden in this particular population. The decision reflects the view of the steering committee. The consensus meeting allows for a further opportunity to discuss, validate and confirm the final. Wording to this effect has been added to the consensus meeting section of the methods.
(12) Dissemination: Defining COS is good, but please add that outcome measurement instruments need to be developed next. This project will identify the concepts/domains only.	We agree that it is important to emphasize that only the outcomes are identified, and that instruments to assess these outcomes (appropriately) should be identified in later stages.<br>Wording to this effect has now been added to the ethics and dissemination section.
(13) Abstract: Please adjust accordingly. Don't say "This paper presents..." Instead describe the objectives.	Thank you, this has now been re-worded.

For peer review only

BMJ Open

Development of a Core Outcome Set and Identification of Patient-Reportable Outcomes for Primary Brain Tumour Trials: Protocol for the COBra Study

Journal:	*BMJ Open*
Manuscript ID	bmjopen-2021-057712.R2
Article Type:	Protocol
Date Submitted by the Author:	12-Jul-2022
Complete List of Authors:	Retzer, Ameeta; University of Birmingham, Centre for Patient Reported Outcomes Research, Institute for Applied Health Research; National Institute for Health and Care Research (NIHR), Applied Research Centre, West Midlands<br>Sivell, Stephanie; Cardiff University, Marie Curie Palliative Care Research Centre, Division of Population Medicine, Cardiff University School of Medicine, College of Biomedical and Life Sciences<br>Scott, Hannah; University of Cambridge, Cambridge Public Health, University of Cambridge School of Clinical Medicine<br>Nelson, Annmarie; Cardiff University School of Medicine, Marie Curie Palliative Care Research Centre, Division of Population Medicine, Cardiff University School of Medicine, College of Biomedical and Life Sciences<br>Bulbeck, Helen; brainstrust<br>Seddon, Kathy; Cardiff University<br>Grant, Robin ; Department of Clinical Neurosciences, Royal Infirmary of Edinburgh<br>Adams, Richard; University of Cardiff, Centre for Trials Research<br>Watts, Colin; University of Birmingham, Institute of Cancer and Genomic Sciences, College of Medical and Dental Sciences<br>Aiyegbusi, Olalekan Lee; University of Birmingham, Centre for Patient Reported Outcomes Research, Institute of Applied Health Research; University of Birmingham, NIHR Birmingham Biomedical Research Centre, University of Birmingham and University Hospitals Birmingham NHS Foundation Trust<br>Kearns, Pamela; University of Birmingham, Institute of Cancer and Genomic Sciences, College of Medical and Dental Sciences; University of Birmingham, NIHR Birmingham Biomedical Research Centre<br>Cruz Rivera, Samantha; University of Birmingham, Centre for Patient Reported Outcomes Research, Institute of Applied Health Research; University of Birmingham, Birmingham Health Partners Centre for Regulatory Science and Innovation<br>Dirven, Linda; Leiden University, Department of Neurology, Leiden University Medical Center; Medical Centre Haaglanden, Department of Neurology<br>Baddeley, Elin; Cardiff University, Marie Curie Palliative Care Research Centre, Division of Population Medicine, Cardiff University School of Medicine, College of Biomedical and Life Sciences<br>Calvert , Melanie ; University of Birmingham, Centre for Patient Reported Outcomes Research, Institute of Applied Health Research; National

	Institute for Health and Care Research (NIHR), Applied Research Centre West Midlands<br>Byrne, Anthony; Cardiff University, Marie Curie Palliative Care Research Centre, Division of Population Medicine, Cardiff University School of Medicine, College of Biomedical and Life Sciences
**Primary Subject Heading**:	Oncology
Secondary Subject Heading:	Palliative care, Patient-centred medicine, Qualitative research, Research methods
Keywords:	Neurological oncology < NEUROLOGY, Neurological oncology < ONCOLOGY, Adult palliative care < PALLIATIVE CARE, QUALITATIVE RESEARCH, Clinical trials < THERAPEUTICS

SCHOLARONE™
Manuscripts

Development of a Core Outcome Set and Identification of Patient-Reportable Outcomes for Primary Brain Tumour Trials: Protocol for the COBra Study

Authors: Ameeta Retzer[1,2,3], Stephanie Sivell[4], Hannah Scott[5], Annmarie Nelson[4], Helen Bulbeck[6], Kathy Seddon[7], Robin Grant[8], Richard Adams[9], Colin Watts[10], Olalekan Lee Aiyegbusi[1,2,3,11,12], Pamela Kearns[10,12, 13], Samantha Cruz Rivera[1,2,11], Linda Dirven[14,15], Elin Baddeley[4], Melanie Calvert[1,2,3,11,12,16, 17] , Anthony Byrne[4]

[1]Institute of Applied Health Research, University of Birmingham, Birmingham, UK

[2]Centre for Patient Reported Outcomes Research, Institute of Applied Health Research, University of Birmingham, Birmingham, UK

[3]National Institute for Health and Care Research (NIHR) Applied Research Centre West Midlands, Birmingham, UK

[4] Marie Curie Palliative Care Research Centre, Division of Population Medicine, Cardiff University School of Medicine, College of Biomedical and Life Sciences, UK

[5] Cambridge Public Health, University of Cambridge School of Clinical Medicine, UK

[6] Brainstrust - the brain cancer people, UK

[7]Cardiff University, UK

[8] Department of Clinical Neurosciences, Royal Infirmary of Edinburgh, UK

[9] Centre for Trials Research, Cardiff University, UK

[10] Institute of Cancer and Genomic Sciences, College of Medical and Dental Sciences, University of Birmingham, UK

[11]Birmingham Health Partners Centre for Regulatory Science and Innovation, University of Birmingham, Birmingham, UK

[12]NIHR Birmingham Biomedical Research Centre, University of Birmingham, Birmingham, UK

[13] Cancer Research UK Clinical Trials Unit (CRCTU), Institute of Cancer and Genomic Sciences, University of Birmingham, UK[14] Department of Neurology, Leiden University Medical Center, Leiden, The Netherlands

[15] Department of Neurology, Haaglanden Medical Center, The Hague, The Netherlands

For peer review only - http://bmjopen.bmj.com/site/about/guidelines.xhtml

[16]NIHR Surgical Reconstruction and Microbiology Research Centre, University of Birmingham, Birmingham, UK

[17]Midlands Health Data Research UK, Birmingham, UK

Correspondence to: Professor Anthony Byrne (anthony.byrne2@wales.nhs.uk)

Abstract:

Introduction

Primary brain tumours, specifically gliomas, are a rare disease group. The disease and treatment negatively impacts on patients and those close to them. The high rates of physical and cognitive morbidity differ from other cancers causing reduced health-related quality of life. Glioma trials using outcomes that allow holistic analysis of treatment benefits and risks enable informed care decisions. Currently, outcome assessment in glioma trials is inconsistent, hindering evidence synthesis. A core outcome set (COS): an agreed minimum set of outcomes to be measured and reported may address this. International initiatives focus on defining core outcomes assessments across brain tumour types. This protocol describes the development of a COS for use in glioma trials, applicable across glioma types involving UK stakeholders, with provision to identify subsets as required. Due to stakeholder interest in data reported from the patient perspective, outcomes from the COS that can be patient-reported will be identified.

Methods and analysis

Stage I: (i) trial registry review to identify outcomes collected in glioma trials and (ii) systematic review of qualitative literature exploring glioma patient and key stakeholder research priorities. Stage II: semi-structured interviews with glioma patients and caregivers. Outcome lists will be generated from Stages I and II. Stage III: study team will remove duplicate items from the outcome lists and ensure accessible terminology for inclusion in the Delphi survey. Stage IV: a two-round Delphi process whereby the outcomes will be rated by key stakeholders. Stage V: a consensus meeting where participants will finalise the COS. The study team will identify the COS outcomes that can be patient-reported. Further research is needed to match patient-reported outcomes to available measures.

Ethics and dissemination

Ethical approval was obtained (REF SMREC 21/59, Cardiff University School of Medicine Research Ethics Committee). Study findings will be disseminated widely through conferences and journal publication. The final COS will be adopted and promoted by patient and carer groups and its use by funders encouraged.

Trial and PROSPERO registration

Core Outcome Measures in Effectiveness Trials (https://www.comet-initiative.org/Studies/Details/1793); PROSPERO (CRD42021236979).

For peer review only - http://bmjopen.bmj.com/site/about/guidelines.xhtml

Strengths and Limitations

- This study collects original qualitative data to ensure all outcomes prioritised by glioma patients are identified. However, this is a resource-intensive process that may not be available to all core outcome set developers.
- Review of trial registries represents a pragmatic approach to comprehensively identify outcomes used in trials rather than reliance on often incomplete outcome reporting in glioma trial publications[1]. There are limitations to this approach - use of trial registries means those that are not registered will not be identified, registry use is inconsistent globally, completeness and specificity can be questionable, and updating of entries continues to be a challenge[2]. However, the quality of registration has been observed to be improving and trial registration associated with subsequent publication and use of the same outcomes as defined in their protocols as in their published reports[3].
- Bias may be introduced by inviting qualitative interview participants from Stage II to take part in Delphi; though this encourages familiarity with concepts, enabling meaningful participation in the Delphi
- Qualitative data collected from the UK population may limit international applicability, though this allows exploration of issues that may be specific to UK context and validation of this COS for use in other settings should be explored.

Introduction:

Primary brain tumours, specifically gliomas, are part of a rare disease group[4]. The disease and its treatment have negative effects on patients and those close to them. The high rates of physical and cognitive morbidity differ from other cancers, with significant impact on a wide range of functional domains. Gliomas are the commonest form of primary brain tumour[5], accounting for 80% of malignant brain tumours. Gliomas represent a heterogeneous group of cancers with variable outcome, traditionally graded from I to IV (least to most aggressive) [2]. However, rapid developments in molecular diagnostics have led to refinements in nomenclature, suggesting a more nuanced approach to brain tumours classification[6]. This would acknowledge the spectrum ranging from a variable but slower-progressing course, such as oligodendroglioma or astrocytoma, to fast-growing tumours such as glioblastoma, a particularly aggressive subtype with a median survival of 12 to 15 months and 5% five year survival rate[7].

The poor prognosis of some glioma patients and the high symptom burden has led to a growing emphasis on their quality of survival[8]. Maintaining cognitive function, physical function and other health-related quality of life aspects throughout the disease trajectory are key considerations alongside very modest survival benefits captured through traditional metrics of tumour response and overall or progression-free survival, particularly for patients with aggressive forms of glioma [9]. Therefore, it is important that glioma intervention studies collect a range of data aligned with patient priorities to enable assessment of the net clinical benefit of treatments [10-13].

Data collected to evidence effects of interventions are known as “outcomes”. Outcomes include traditional measures such as progression-free survival and radiological tumour response but also Clinical Outcome Assessments (COAs). COAs describe how a patient feels,

For peer review only - http://bmjopen.bmj.com/site/about/guidelines.xhtml

functions, or survives. COAs include Clinician Reported Outcomes (ClinRO), Observer Reported Outcomes (ObsRO), Performance Outcomes (PerfO), and Patient Reported Outcomes (PROs)[14]. PROs assess a range of outcomes including symptoms, functional health, well-being and psychological issues from the patients' perspective, without interpretation by a clinician or anyone else [15]. When assessing treatments, PROs enable insight into the impact of treatment on patient's perceived wellbeing where other outcome data that may indicate minimal differences in disease control and survival, potentially influencing patients' treatment choices [16].

Interpreting the clinical benefit of treatments requires effective data synthesis and meta-analyses of trial outcomes. This requires consistent use of outcomes, use of appropriate outcome measures, and diligent data capture, analysis and reporting. Inconsistent outcome use is widespread. A significant lack of standard ontology has been found in cancer clinical trials [17] and in brain tumour studies specifically[18]. Moreover, selective outcome and missing data reporting is common[19], introducing bias and hindering evidence synthesis. PROs are critical to the comprehensive evaluation of treatment benefits and side effects, and are increasingly used by regulatory authorities. The Food and Drug Administration (FDA) is prioritising a patient-centred approach to drug development[20], a consistent approach to PRO use generally [21], and in cancer clinical trials specifically[22].The European Medicines Agency (EMA) support PRO use to assess drug efficacy and tolerability in informing product approval in cancer[23], consistent with the FDA[24, 25]. Key PROs for use in cancer has been of consistent interest[22, 26, 27], patients value this form of data [28-31], and it underpins informed shared decision-making [32-35]. However, there is limited consensus on which areas of patient experience should be consistently assessed in brain tumour trials. In cancer trials using PROs, analyses are often unreported in publications and the clinical relevance of PRO results are overlooked [36]. A systematic review of glioma randomised controlled trials (RCTs) using PROs found that only 14% of these trials met the criteria for high quality reporting [37], with PRO results not being interpreted in 79%, and clinical relevance not discussed in 86% of trials.

There are international efforts to unify and improve practice. In PRO research in the field of neuro-oncology, the Response Assessment in Neuro-Oncology Patient Reported Outcomes (RANO-PRO) working group aims to provide guidance on Patient-Reported Outcome Measures (PROMs) in adult neuro-oncology clinical trials and practice (23). Their systematic review (26) found that 215 PROs have been used in brain tumour (primary and secondary) studies, the majority only used once or twice. The FDA and EMA recognise the importance of assessing symptoms, adverse effects and function as core constructs in all glioma trials[38], and have participated in an international multi-stakeholder workshop aiming to define a core set of priority constructs to be assessed as minimum in high grade glioma trials and care[39].

Core Outcome Sets (COS) establish 'the minimum that should be measured and reported in all clinical trials of a specific condition' [40], aiming to achieve consensus between researchers, clinicians, patients and policy makers. This facilitates consistent outcome collection, analysis, and reporting, enables data synthesis and meta-analyses, reduces research waste, and informs patient-centred care. Upon COS confirmation, further research will determine how to measure these outcomes.

For peer review only - http://bmjopen.bmj.com/site/about/guidelines.xhtml

The primary aim of this research is to develop a COS for use in adult primary glioma (astrocytoma, oligodendroglioma, oligoastrocytoma, ependymoma, astroblastoma, anaplastic ganglioglioma, glioblastoma, glioblastoma multiforme) phase III interventional trials comprising all outcome types. We will define outcomes applicable to all glioma as well those that may be specific to glioma types. The COS will inform interpretation of the net clinical benefit of interventions in terms that reflect stakeholder priorities. Due to interest in core PROs in cancer, our secondary aim is to identify the COS outcomes which can be patient reported.

Focus of COS
This COS will apply to phase III interventional trials for systemic anti-cancer treatments (including immunotherapy and chemotherapy), radiotherapy, surgery, and supportive care involving adults (aged over 18 years), diagnosed with glioma, with a specific focus on the UK population. Though some data formulating this COS will be drawn from a UK sample, trialists should consider the COS to be applicable internationally. To promote generalisability of results, recruitment into the qualitative interviews and Delphi exercise will be monitored for glioma type, age, ethnicity, and gender.

Methods and analysis

Objectives:

1. Trial registry review to identify glioma trial outcomes and a systematic review of the qualitative literature to explore key stakeholders' research and treatment priorities;
2. Identify outcomes using qualitative interviews with glioma patients and caregivers;
3. Combine the results of Objectives 1 and 2 into a unified longlist of outcomes;
4. Achieve consensus on a COS through online Delphi process and a consensus meeting with a range of stakeholders.

Study Design
The COBra (Patient Reported Core Outcomes in Brain Tumour Trials) study uses a mixed-methods, multi-stage approach in accordance with accepted COS methodology [41] and guidance[42] (Appendix 1) and registered with the Core Outcome Measures in Effectiveness Trials (COMET) Initiative[43].

Study Team and Collaborators
The study team is multidisciplinary, including Patient and Public Involvement (PPI) representatives, healthcare professionals, researchers, policy makers, and regulators.

The Marie Curie Palliative Care Research Centre Cardiff, the Centre for Patient Reported Outcomes Research, the Centre for Trials Research (Cardiff University) and Birmingham Clinical Trials Unit (CTU) will provide methodological steer on behalf of the Supportive and Palliative Care subgroup of the NCRI (National Cancer Research Institute) Brain Tumour group. Collaboration with the RANO-PRO Initiative[44] working group will ensure alignment with international efforts.

For peer review only - http://bmjopen.bmj.com/site/about/guidelines.xhtml

Patient and Public Involvement

The PPI team members contributed to study design and will develop and monitor the study as part of the Steering Group[45], contributing to data analysis and dissemination of study findings. The study team will seek advice from a wider panel of PPI representatives convened for the purpose of the study, consisting of individuals with a range of backgrounds and experiences. The detailed participation of PPI representatives will be reported in accordance with GRIPP2 (Guidance for Reporting Involvement of Patients and the Public) [46].

Stage I – Evidence review

Aims

Review of clinical trial registries and a systematic review of published qualitative literature to generate an outcome list [41] from:

A) Phase III interventional glioma trials involving adult patients and diagnosed with primary glioma;
B) Qualitative studies exploring the lived experience and research priorities of adult patients with primary glioma, and other key stakeholders.

Search strategy and data extraction

Search A

ClinicalTrials.gov and ISRCTN clinical trials registries, based in the US and UK respectively, will be used to identify outcomes used in phase III interventional glioma trials in adults (Appendix 2). Data from both are available for public download. Where protocols are available alongside registration information, these will be retrieved.

Two reviewers will independently perform complete searches of glioma trials registered on clinicaltrials.gov and isrctn.com without restriction by date. The results will be independently reviewed for eligibility; disagreements will be resolved with a third reviewer. Two reviewers will independently extract data including basic trial information, year of study, primary outcome(s) and secondary outcomes. Data in the csv files will be cross-referenced with clinical trial registration entry for completeness, and with the protocol when available. The most recently updated of these will be used.

Trials sourced during Search A will be cross-referenced with those retrieved from the RANO-PRO study for information.

Search B

We will systematically review the qualitative literature describing the experiences and needs of adults diagnosed with glioma and thematically synthesise (44) their 'lived experiences' in relation to care, treatment and treatment outcomes.

For peer review only - http://bmjopen.bmj.com/site/about/guidelines.xhtml

Databases to be searched include MEDLINE, EMBASE, CINAHL, Web of Science, PsycINFO, the Cochrane Central Register of Controlled Trials and the Cochrane Library. Reference lists of key authors and journals will be hand searched. Qualitative studies, or mixed-method studies containing qualitative data, published in the English language, restricted to 15 years prior, will be included. This is because of limited data prior and literature captured is more reflective of current treatment options and patient perspective Research involving adult patients and/or key stakeholders including informal carergivers, will be included. Two reviewers will independently review all titles and abstracts; a third reviewer will review citations for any disagreements. Full text studies will be reviewed by two reviewers; disagreements will be resolved with a third reviewer.

Two reviewers will independently extract data using a standardised data collection form, capturing the themes and sub-themes of the qualitative data pertaining to the lived experience of patients with primary glioma. The qualitative literature will be thematically synthesised following three stages: coding text, developing descriptive themes and generating themes[47]. The data will focus on patients and key stakeholders including informal carergivers, exploring their interpretation of patients' 'lived experiences', including views relating to their attitudes and experience of symptoms and functional outcomes. NVivo[48] will be used for data management.

Stage II – Interviews with patients and caregivers
Semi-structured interviews will be conducted with adults diagnosed with primary glioma across the spectrum of the disease. Interview participants can identify a caregiver to join them in an interview dyad. The interviews will inform the language used in the Delphi survey and identify outcomes not captured during Stage I.

Aims
The objectives of these interviews are to explore:

(i) outcomes that are important to patients;
(ii) caregivers' understanding of patients' priorities and experiences, as these may differ.

Participant eligibility and sampling
Dyads will comprise eligible patients histologically diagnosed with primary glioma (astrocytoma, oligodendroglioma, oligoastrocytoma, ependymoma, astroblastoma, anaplastic ganglioglioma, glioblastoma, glioblastoma multiforme) and a caregiver identified by the patient. Caregivers are defined as informal carers, who may be a family member or friend, who provides the majority of the support to the patient and is able to estimate the patient's priorities. Patients and caregivers will be over the age of 18 years.

Participants will be recruited through the NCRI Brain Group, the Tessa Jowell BRAIN MATRIX trial platform[49], CTUs, brainstrust – the brain cancer people, The Brain Tumour Charity, snowballing, known contacts, and social media platforms. Potential participants will be invited to contact the research team to express interest. Recruitment will be monitored to promote diversity in terms of glioma type, age, ethnicity, and gender, seeking balance between glioma types. Between 12 and 20 dyads representing the spectrum of malignant disease will be recruited based on previous studies and expected data saturation[50]. Data

For peer review only - http://bmjopen.bmj.com/site/about/guidelines.xhtml

saturation will be assessed through constant discussion and evaluation of the data by the qualitative researchers conducting the data collection and analysis, together with members of the wider study team. Recruitment will end when data saturation is reached.

Consent and Capacity

Patients and caregivers will give consent on their own behalf if they wish to participate in an interview. If a patient or caregiver does not proceed with an interview, the other will still be invited to participate. Their permission is not required for the other to participate. Information sheets will be sent to eligible participants via post or email with the contact details of the research team member conducting the interviews. Participants expressing interest will be given the chance to ask any questions prior to consent. Participants will complete an electronic or hardcopy consent form or will be recorded giving verbal consent, depending on interview format.

In accordance with the Mental Capacity Act (2005), patient participants will be assumed to have capacity unless it is proven otherwise. If there is concern that the patient lacks capacity to participate, this will be discussed with the Chief Investigator, a clinician, about whether further research activity will occur. If research will not continue with the patient participant, the caregiver will be given the opportunity to take part in an interview to share their views.

Data collection

A semi-structured interview format will be used to understand patient experiences of living with glioma, and what they consider to be the most important outcomes from glioma treatment. Caregiver participants' perspective of patients' experience and priorities will be captured, not a direct report of the patients' condition. The interviews will be undertaken via phone or video link (e.g. Zoom or Microsoft Teams), or face-to-face, depending on the situation and preference of patients. Interviews may take place with patients and caregivers together or separately, depending on their preference. Interviews where patients and caregivers are interviewed separately allow for differing views to be expressed. Where interviews are undertaken together, efforts will be made to ensure both are able to express their views. Interviews will be audio-recorded. The interview will be guided by open-ended questions on diagnosis, treatment, and their effects on patients and caregivers, directed towards understanding outcomes important to patients. The semi-structured format allows for spontaneous exploration of novel topics. The topic-guide may be reviewed and adapted iteratively after the first few interviews, if required. At the end of the interviews, participants will be asked directly which outcomes they believe should be measured in clinical trials. This places the lived experience of participants at the forefront, with patients and caregivers given the chance to talk about the things that matter most to them.

Data Analysis

The interview data, once transcribed and anonymised, will be thematically analysed [51] using NVivo software [48] for data management. A preliminary framework will be derived from the available literature including the Thematic analysis allows for the identification of patterns and themes within the data, to organise and describe data in rich detail[51]. It is particularly well-suited to studies that focus on lived experience. Data collected from patients and caregivers will be analysed and formulated into separate accounts.

For peer review only - http://bmjopen.bmj.com/site/about/guidelines.xhtml

Analysis of the first three transcripts will be conducted independently by two members of the research team experienced in qualitative research and a draft coding structure will be formulated. Disagreements in coding will be resolved through discussion and input from a third qualitative researcher will be sought when required. The draft coding frame will be reviewed by PPI team members and a coding structure for the remaining transcripts will be confirmed. The framework will be refined, until the analysis of all transcripts has been completed, with the findings synthesised into categories and subcategories.

Stage III – Review of outcome list
All outcomes, without limitation by outcome type, captured in Stage I will be grouped and classified [41]. A broad ontology for this will be developed from the framework outlined in the COMET handbook and relevant frameworks from the available literature[38] in advance of outcome extraction and will be iteratively refined based on the outcomes identified. The ontology will serve as a categorical tool to organise and present the outcomes in an accessible manner. Each grouping will contain domains and subdomains that broadly measure particular aspects of the effects of interventions (e.g. symptoms and function)[52]. The outcome lists formed by each of the two researchers will be compared for completeness, and differences in the categorisation will be resolved through discussion.

The categories and subcategories generated in Stage II will be formulated into an outcome list and differences in the categorisation will be resolved through discussion.

A longlist of outcomes will be generated from the Stage I and II outcome lists. Duplicates will be removed during this process. This list will be reviewed by the study team to refine the language used to describe the outcomes. The team will review the structure of the questions included in the Delphi survey. At this stage, it will be decided whether separate Delphi processes are needed according to glioma type based on the emerging data.

Stage IV – Delphi survey

A modified two-round Delphi will be used to assess the relative importance of outcomes included in the stage III outcome list. Participants will be invited to consider applicability of the COS to new and emerging therapies, and whether the outcomes would apply. The aim of the Delphi process is to reach consensus on which outcomes should form the COS for glioma trials.

Recruitment
Approximately 100 participants with professional or personal experience of glioma care and treatment: 1) patients, 2) caregivers, 3) healthcare professionals and researchers, 4) policy-makers and regulators will be recruited as previously described in earlier stages. During Delphi registration participants will choose the stakeholder group with which they most identify but can note if they identify with other stakeholder groups besides their primary. Approximately 25 participants will be recruited to each stakeholder group, recruitment will be monitored and will inform and direct efforts as required. Consent will be taken electronically during the online registration process.

Delphi process

For peer review only - http://bmjopen.bmj.com/site/about/guidelines.xhtml

The Delphi exercise will reflect COMET recommendations [41] and will present the Stage III outcome list. Participants will rate each of the outcomes on a 9-point Likert scale, (1–3, not important; 4–6, important but not critical; and 7–9, important and critical)[53]. During Round 1, participants can add outcomes they feel are missing. Votes from individuals in each stakeholder group will be given equal weighting. All original outcomes will be presented in Round 2. Outcomes added by participants in Round 1 will be presented in Round 2. In Round 2, respondents will be presented with their own rating for each outcome and how it was rated by their own stakeholder group. Based on this information, respondents will be invited to amend their score, if they wish. During Round 2, participants can rate the outcomes suggested in Round 1.

The threshold for consensus for inclusion in or exclusion from the COS will be ≥70%, informed by those used in comparable COS development studies [54, 55]. After the Delphi, outcomes will be proposed for inclusion in the final COS if ≥70% respondents rate the item as 7-9 and ≤15% rate the item as 1-3. Items will be proposed for exclusion from the final COS if ≥70% respondents rate the item as 1-3 and ≤15% rate the item as 7-9. Those outcomes that do not reach agreement after the two Delphi rounds will be discussed in the consensus meeting, together with the items proposed for inclusion and exclusion.

Missing data
To minimise partial response, participants will be unable to skip questions but can indicate when they feel unable to rank specific items. Reminders will be used to minimise participant attrition between Delphi rounds. Use of specialised Delphi software, Delphi Manager, will enable rapid inter-round rating calculations to allow the second round to open with minimal delay to further reduce attrition.

Stage V consensus meeting
This meeting may be held virtually or in person, depending on the situation and preference of the majority of participants. All Delphi participants will be invited. Notes will be taken during the meeting and consent will be sought from all participants to audio-record the meeting for reference. Decisions made during the consensus meeting will be made through anonymous voting using voting software. Decisions will proceed if ratified by ≥70% of the group. In cases where there is <100% consensus, decisions will be discussed until those in disagreement are satisfied that their views have been considered and that the decision can proceed. This meeting allows for a further opportunity to discuss, validate and the confirm the final COS. The core outcomes applicable to all glioma trials will be agreed, as will any outcomes identified as specific to particular types of glioma. Following the consensus meeting, the study team will identify which of the outcomes could be assessed by patient reporting.

Ethics and dissemination
Ethical approval was granted (REF: SMREC 21/59, Cardiff University School of Medicine Research Ethics Committee). All data will be collected and stored in accordance with local regulations[56].

For peer review only - http://bmjopen.bmj.com/site/about/guidelines.xhtml

The final COS will be published in compliance with accepted reporting standards [41] and adopted and promoted by the NCRI Brain Clinical Studies Group Supportive and Palliative Care subgroup for use in glioma studies. The subgroup will publish a position statement mandating for UK CTUs involved in brain tumour research to implement the COS.
Study findings will be disseminated widely, including to national and international conferences and high-impact journals. A plain English summary will be co-produced with PPI team members and made available to participants upon request. The COS will be promoted amongst patient and carer groups using The Brain Tumour Charity network (including BRIAN), NCRI and regional PPI frameworks, brainstrust, and other patient organisations. The importance of COS development is increasingly recognised by funders, such as the National Institute for Health and Care Research, and regulators, such as EMA and FDA. The COS will therefore be promoted to encourage its inclusion in 'justification of outcomes' sections of funding proposals and regulatory submissions. The final COS will be freely available on the COMET database.

Though the participants in the original qualitative data collection will be drawn from a UK sample and the Delphi participants will be largely based in the UK, the trial registry searches were without restriction based on country and the qualitative literature were limited to those in English language only. The study steering committee has membership from stakeholders leading international initiatives and the Delphi survey and consensus meeting will involve participants from international regulatory bodies. As a result, the resulting COS should be considered to be internationally applicable. For use in other settings or countries, validation exercises are advised to ensure economic and cultural differences are integrated. The study team will consider the findings of this study in the context of existing international initiatives. Findings will be shared with international partners and may be integrated into international guidance on outcome assessment across all brain tumour types.
COBra will directly collaborate with the RANO-PRO working group and affiliated international initiatives. Following study completion, RANO-PRO findings may be used to select appropriate COAs aligned to the COS. COBra will also collaborate with UK funders, trialists and CTUs on COS implementation and the consistent application of international standards for collection, analysis and reporting of the COS across all UK studies.

Following finalising the COS, further research is required to identify and/or develop corresponding outcome measures.

References

1. Tardy, M.P., et al., *Quality of Randomized Controlled Trials Reporting in the Treatment of Adult Patients with High-Grade Gliomas.* Oncologist, 2018. **23**(3): p. 337-345.
2. Viergever, R.F. and K. Li, *Trends in global clinical trial registration: an analysis of numbers of registered clinical trials in different parts of the world from 2004 to 2013.* BMJ Open, 2015. **5**(9): p. e008932.

For peer review only - http://bmjopen.bmj.com/site/about/guidelines.xhtml

3. Chan, A.-W., et al., *Association of Trial Registration With Reporting of Primary Outcomes in Protocols and Publications.* JAMA, 2017. **318**(17): p. 1709-1711.
4. Diseases, N.O.f.R. *Glioma*. 2019 [cited 2021 21 September].
5. Goodenberger, M.L. and R.B. Jenkins, *Genetics of adult glioma.* Cancer Genet, 2012. **205**(12): p. 613-21.
6. Wood, M.D., A.M. Halfpenny, and S.R. Moore, *Applications of molecular neuro-oncology - a review of diffuse glioma integrated diagnosis and emerging molecular entities.* Diagnostic Pathology, 2019. **14**(1): p. 29.
7. UK, C.R. *Survival*. 2019 [cited 2021 1 June].
8. Sagberg, L.M., O. Solheim, and A.S. Jakola, *Quality of survival the 1st year with glioblastoma: a longitudinal study of patient-reported quality of life.* Journal of Neurosurgery JNS, 2016. **124**(4): p. 989-997.
9. Basch, E., et al., *Patient-Reported Outcomes in Cancer Drug Development and US Regulatory Review: Perspectives From Industry, the Food and Drug Administration, and the Patient.* JAMA Oncol, 2015. **1**(3): p. 375-9.
10. MacDonald, L.o.b.o.t.N.-O.G. *Top 10 priorities for clinical research in primary brain and spinal cord tumours: final report of the James Lind Alliance Priority Setting Partnership in Neuro-Oncology.* 2015 28 January 2021].
11. Collins, A., et al., *The challenges and suffering of caring for people with primary malignant glioma: qualitative perspectives on improving current supportive and palliative care practices.* BMJ Support Palliat Care, 2014. **4**(1): p. 68-76.
12. Piil, K., et al., *Controlled rehabilitative and supportive care intervention trials in patients with high-grade gliomas and their caregivers: a systematic review.* BMJ Support Palliat Care, 2016. **6**(1): p. 27-34.
13. Armstrong, T.S. and M.R. Gilbert, *Net clinical benefit: functional endpoints in brain tumor clinical trials.* Curr Oncol Rep, 2007. **9**(1): p. 60-5.
14. Administration, U.F.a.D. *Clinical Outcome Assessment (COA) Qualification Program*. 2021 [cited 2021 1 June].
15. FDA. *Guidance for industry: Patient-Reported outcome measures: Use in medical product development to support labeling claims*. 2009 [cited 2021 2 June].
16. Fairclough, D.L., *Patient reported outcomes as endpoints in medical research.* Statistical Methods in Medical Research, 2004. **13**(2): p. 115-138.
17. Hirsch, B.R., et al., *Characteristics of oncology clinical trials: insights from a systematic analysis of ClinicalTrials.gov.* JAMA Intern Med, 2013. **173**(11): p. 972-9.
18. Dirven, L., et al., *Systematic review on the use of patient-reported outcome measures in brain tumor studies: part of the Response Assessment in Neuro-Oncology Patient-Reported Outcome (RANO-PRO) initiative.* Neuro-Oncology Practice, 2021.
19. Kirkham, J.J., et al., *Can a core outcome set improve the quality of systematic reviews?--a survey of the Co-ordinating Editors of Cochrane Review Groups.* Trials, 2013. **14**: p. 21.
20. Administration, F.a.D. *FDA Patient-Focused Drug Development Guidance Series for Enhancing the Incorporation of the Patient's Voice in Medical Product Development and Regulatory Decision Making*. 2020 [cited 2021 3 August].
21. *Guidance for industry: patient-reported outcome measures: use in medical product development to support labeling claims: draft guidance.* Health Qual Life Outcomes, 2006. **4**: p. 79.
22. Administration, F.a.D. *Core Patient-Reported Outcomes in Cancer Clinical Trials: Draft Guidance for Industry*. 2021 [cited 2021 3 August].
23. Agency, E.M. *Appendix 2 to the guideline on the evaluation of anticancer medicinal products in man : The use of patient-reported outcome (PRO) measures in oncology studies*. 2016 [cited 2021 3 August].

For peer review only - http://bmjopen.bmj.com/site/about/guidelines.xhtml

24. Basch, E. and C. Yap, *Patient-Reported Outcomes for Tolerability Assessment in Phase I Cancer Clinical Trials.* JNCI: Journal of the National Cancer Institute, 2021. **113**(8): p. 943-944.
25. Kluetz, P.G., et al., *Informing the Tolerability of Cancer Treatments Using Patient-Reported Outcome Measures: Summary of an FDA and Critical Path Institute Workshop.* Value Health, 2018. **21**(6): p. 742-747.
26. Reeve, B.B., et al., *Recommended Patient-Reported Core Set of Symptoms to Measure in Adult Cancer Treatment Trials.* JNCI: Journal of the National Cancer Institute, 2014. **106**(7).
27. Kluetz, P.G., et al., *Focusing on Core Patient-Reported Outcomes in Cancer Clinical Trials: Symptomatic Adverse Events, Physical Function, and Disease-Related Symptoms.* Clin Cancer Res, 2016. **22**(7): p. 1553-8.
28. Calvert, M.J. and N. Freemantle, *Use of health-related quality of life in prescribing research. Part 1: why evaluate health-related quality of life?* J Clin Pharm Ther, 2003. **28**(6): p. 513-21.
29. Ouwens, M., et al., *Development of indicators for patient-centred cancer care.* Support Care Cancer, 2010. **18**(1): p. 121-30.
30. Brundage, M., et al., *Cancer patients' preferences for communicating clinical trial quality of life information: a qualitative study.* Qual Life Res, 2003. **12**(4): p. 395-404.
31. Davison, B.J., et al., *Provision of individualized information to men and their partners to facilitate treatment decision making in prostate cancer.* Oncol Nurs Forum, 2003. **30**(1): p. 107-14.
32. England, N. *Shared decision making*. 2021 [cited 2021 1 June].
33. Excellence, N.I.f.H.a.C. *Shared decision making*. 2021 [cited 2021 1 June].
34. Au, H.J., et al., *Added value of health-related quality of life measurement in cancer clinical trials: the experience of the NCIC CTG.* Expert Rev Pharmacoecon Outcomes Res, 2010. **10**(2): p. 119-28.
35. Basch, E., *Toward patient-centered drug development in oncology.* N Engl J Med, 2013. **369**(5): p. 397-400.
36. Pe, M., et al., *Statistical analysis of patient-reported outcome data in randomised controlled trials of locally advanced and metastatic breast cancer: a systematic review.* Lancet Oncol, 2018. **19**(9): p. e459-e469.
37. Dirven, L., et al., *The level of patient-reported outcome reporting in randomised controlled trials of brain tumour patients: a systematic review.* Eur J Cancer, 2014. **50**(14): p. 2432-48.
38. Armstrong, T.S., et al., *Glioma patient-reported outcome assessment in clinical care and research: a Response Assessment in Neuro-Oncology collaborative report.* The Lancet Oncology, 2020. **21**(2): p. e97-e103.
39. Helfer, J.L., et al., *Report of the Jumpstarting Brain Tumor Drug Development Coalition and FDA clinical trials clinical outcome assessment endpoints workshop (October 15, 2014, Bethesda MD).* Neuro-oncology, 2016. **18 Suppl 2**(Suppl 2): p. ii26-ii36.
40. Kirkham, J.J., et al., *Core Outcome Set-STAndards for Development: The COS-STAD recommendations.* PLoS Med, 2017. **14**(11): p. e1002447.
41. Williamson, P.R., et al., *The COMET Handbook: version 1.0.* Trials, 2017. **18**(Suppl 3): p. 280.
42. Kirkham, J.J., et al., *Core Outcome Set-STAndardised Protocol Items: the COS-STAP Statement.* Trials, 2019. **20**(1): p. 116.
43. Byrne A, S.S., Scott H, Nelson A, Seddon K, Calvert M, Retzer A, Grant R, Bulbeck H, Adams R, Watts C, Aiyegbusi OL, Kearns P, Cruz Rivera S. *Patient Reported Core Outcomes in Brain Tumour Trials: the COBra study*. 2020 [cited 2021 5 August].
44. Dirven, L., et al., *Working plan for the use of patient-reported outcome measures in adults with brain tumours: a Response Assessment in Neuro-Oncology (RANO) initiative.* Lancet Oncol, 2018. **19**(3): p. e173-e180.
45. INVOLVE. *UK Standards for Public Involvement: Better public involvement for better health and social care*. 2019 [cited 2021 4 August].

For peer review only - http://bmjopen.bmj.com/site/about/guidelines.xhtml

46. Staniszewska, S., et al., *GRIPP2 reporting checklists: tools to improve reporting of patient and public involvement in research.* Bmj, 2017. **358**: p. j3453.
47. Thomas, J. and A. Harden, *Methods for the thematic synthesis of qualitative research in systematic reviews.* BMC Med Res Methodol, 2008. **8**: p. 45.
48. Ltd, Q.I.P. *NVivo qualitative data analysis software*. 2020 [cited 2021 1 June].
49. Birmingham, U.o. *Tessa Jowell BRAIN MATRIX*. 2020 [cited 2021 5 August].
50. Saunders, B., et al., *Saturation in qualitative research: exploring its conceptualization and operationalization.* Qual Quant, 2018. **52**(4): p. 1893-1907.
51. Braun, V. and V. Clarke, *Using thematic analysis in psychology.* Qualitative Research in Psychology, 2006. **3**(2): p. 77-101.
52. Health, N.I.o. *Patient-Reported Outcomes Measurement Information System–PROMIS*. 2019 [cited 2021 29 January].
53. Guyatt, G.H., et al., *GRADE guidelines: 2. Framing the question and deciding on important outcomes.* J Clin Epidemiol, 2011. **64**(4): p. 395-400.
54. Waters, A.M., et al., *The CONSENSUS study: protocol for a mixed methods study to establish which outcomes should be included in a core outcome set for oropharyngeal cancer.* Trials, 2014. **15**: p. 168.
55. Fish, R., et al., *Core outcome research measures in anal cancer (CORMAC): protocol for systematic review, qualitative interviews and Delphi survey to develop a core outcome set in anal cancer.* BMJ Open, 2017. **7**(11): p. e018726.
56. Department for Digital, C., Media & Sport, Home Office. *Data Protection Act 2018*. 2018 [cited 2021 1 February].

Author contribution

The study concept and design was conceived by AR, SS, HS, AN, HB, KS, RG, RA, CW, OLA, PK, SCR, LD, EB, MC, and AB. MC and AR advised on methodology. EB and AR will undertake the registry review, EB and SS will undertake the qualitative systematic review. EB and SS will recruit, screen and consent participants and will undertake the interviews with input from AR and AB. EB will recruit for the Delphi and consensus meeting, with input from SS, AR, and AB. HS prepared the first draft of the manuscript. AR prepared subsequent drafts. SS, HS, AN, HB, KS, RG, RA, CW, OLA, PK, SCR, LD, EB, MC, and AB all provided edits and critiqued the manuscript for intellectual content.

Funding statement

This study is funded by The Brain Tumour Charity (GN-000704). The funders have no role in study design or manuscript preparation.

Competing interests

Due to their involvement in the study design, the study team members will not participate in the Delphi process or consensus meeting, other than in a facilitative role. Study team members will encourage engagement and participation in the Delphi process and consensus meeting by individuals within the networks of which they are part, as appropriate. AR is partially funded by NIHR Applied Research Collaborative West Midlands. SS is supported by Marie Curie core grant funding to the Marie Curie Palliative Care Research Centre, Cardiff University, grant reference MCC-FCO-11-C. MC is Director of the Birmingham Health Partners Centre for Regulatory Science and Innovation, Director of the Centre for Patient Reported Outcomes Research and is a National Institute for Health Research (NIHR) Senior Investigator. She receives funding from the NIHR Birmingham Biomedical Research Centre, the NIHR Surgical Reconstruction and Microbiology Research Centre and NIHR ARC West Midlands at the at the University of Birmingham and University

For peer review only - http://bmjopen.bmj.com/site/about/guidelines.xhtml

Hospitals Birmingham NHS Foundation Trust, Health Data Research UK, Innovate UK (part of UK Research and Innovation), Macmillan Cancer Support, UCB and GSK Pharma. MC has received personal fees from Astellas, Takeda, Merck, Daiichi Sankyo, Glaukos, GSK, Aparito Ltd, CIS Oncology and the Patient-Centered Outcomes Research Institute (PCORI) outside the submitted work. MC has led the development of SPIRIT-PRO and CONSORT-PRO international guidance and is a member of the SISAQOL and PROTEUS Consortia. OLA receives funding from the NIHR Birmingham Biomedical Research Centre (BRC), NIHR Applied Research Centre (ARC), West Midlands at the University of Birmingham and University Hospitals Birmingham NHS Foundation, Innovate UK (part of UK Research and Innovation), Gilead Sciences Ltd, and Janssen Pharmaceuticals, Inc. OLA declares personal fees from Gilead Sciences Ltd, GlaxoSmithKline (GSK) and Merck outside the submitted work. LD has received research funding (as PI or collaborator) from the Brain Tumour Charity, EORTC Quality of Life Group, Innovative Medicines Initiatives, Dutch Cancer Society, ZonMw, National Institute for Health Research UK, and crowd funding initiatives, although not related to this specific research. LD is involved in the EORTC (as chair of the Brain Tumour Group Quality of Life Committee) and in the development of EORTC questionnaires for brain tumour patients (IADL, BN20). LD is a representative of the RANO PRO initiative. LD is associate Editor for Neuro Oncology, dealing with papers on clinical outcomes in brain tumours.

For peer review only

Appendix 1

		Page
Title 1a	Identify in the title that the paper describes the protocol for the planned development of a COS	1
Abstract 1b	Provide a structured abstract	2
INTRODUCTION Background and objectives 2a	Describe the background and explain the rationale for developing the COS, and identify the reasons why a COS is needed and the potential barriers to its implementation	3-5, 11
2b	Describe the specific objectives with reference to developing a COS	5
Scope 3a	Describe the health condition(s) and population(s) that will be covered by the COS	5
3b	Describe the intervention(s) that will be covered by the COS	5
3c	Describe the context of use for which the COS is to be applied	5
METHODS Stakeholders 4	Describe the stakeholder groups to be involved in the COS development process, the nature of and rationale for their involvement and also how the individuals will be identified; this should cover involvement both as members of the research team and as participants in the study	7-10
Information sources 5a	Describe the information sources that will be used to identify the list of outcomes. Outline the methods or reference other protocols/papers	6-10
5b	Describe how outcomes may be dropped/ combined, with reasons	9-10
Consensus process 6	Describe the plans for how the consensus process will be undertaken	10
Consensus definition 7a	Describe the consensus definition	10
7b	Describe the procedure for determining how outcomes will be added/combined/dropped from consideration during the consensus process	10
ANALYSIS Outcome scoring/ feedback 8	Describe how outcomes will be scored and summarised, describe how participants will receive feedback during the consensus process	10
Missing data 9	Describe how missing data will be handled during the consensus process	10
ETHICS and DISSEMINATION Ethics approval/ informed consent 10	Describe any plans for obtaining research ethics committee/institutional review board approval in relation to the consensus process and describe how informed consent will be obtained (if relevant)	9-11
Dissemination 11	Describe any plans to communicate the results to study participants and COS users, inclusive of methods and timing of dissemination	11
ADMINISTRATIVE INFORMATION Funders 12	Describe sources of funding, role of funders	14
Conflicts of interest 13	Describe any potential conflicts of interest within the study team and how they will be managed	14

For peer review only

For peer review only

Appendix 2

1. ClinicalTrials.gov
Condition/Disease: glioma OR astrocytoma OR oligodendroglioma OR oligoastrocytoma OR ependymoma OR astroblastoma OR anaplastic gangioglioma OR glioblastoma OR GBM OR Glioblastoma multiforme
Study type: Interventional Studies (Clinical Trials)
Age: Adult 18-64 AND Older Adult (65+)
Phase: III

2. ISRCTN.com
Each term searched individually:
Condition/Disease: glioma; astrocytoma; oligodendroglioma; oligoastrocytoma; ependymoma; astroblastoma; anaplastic gangioglioma; glioblastoma; GBM; Glioblastoma multiforme

For peer review only
